# miRNA-182-5p promotes myogenic differentiation of C2C12 cells via the suppression of ZBTB7A

**DOI:** 10.3389/fvets.2025.1637277

**Published:** 2025-07-30

**Authors:** Mengyuan Zhang, Yongheng Wang, Shan Shan, Siyu Liu

**Affiliations:** State Key Laboratory of Reproductive Regulation and Breeding of Grassland Livestock, School of Life Sciences, Inner Mongolia University, Hohhot, China

**Keywords:** miR-182-5p, C2C12, myogenic differentiation, ZBTB7A, dual-luciferase reporter

## Abstract

**Introduction:**

Skeletal muscle possesses a significant regenerative capacity, which is largely mediated by myogenic satellite stem cells. MicroRNAs are known regulators of muscle development. miR-182-5p plays important roles in cell proliferation and migration in various cell types and pathologies. However, its specific role in myogenesis remains unclear. In this study, we elucidated the function of miR-182-5p in the differentiation of C2C12 myoblasts.

**Methods:**

We evaluated the effects of overexpression and inhibition of miR-182-5p in C2C12 cells on its myogenic differentiation ability using Giemsa staining. We also determined the mRNA and protein levels of myogenic differentiation marker genes in these cells at different time points after the induction of differentiation in these cells. The target of miR-182-5p was predicted using bioinformatics tools and validated using luciferase reporter assay.

**Results:**

Overexpression of miR-182-5p via mimic transfection promoted differentiation, while its inhibition by a specific compound attenuated this process. Furthermore, using bioinformatic prediction and validation via a dual-luciferase reporter assay, we identified zinc finger and BTB domain containing 7A (*Zbtb7a*) as a direct target gene of miR-182-5p during C2C12 myogenic differentiation.

**Conclusion:**

Our findings indicate that miR-182-5p positively regulates C2C12 differentiation, partly via the suppression of Zbtb7a and suggest that appropriate miR-182-5p expression is essential for normal myogenesis.

## 1 Introduction

MicroRNAs (miRNAs) are small, endogenous, noncoding RNAs, typically 18–22 nucleotides in length, which function as critical post-transcriptional regulators of gene expression. They exert their effects primarily by binding to the 3′-untranslated regions (3′-UTRs) of target messenger RNAs (mRNAs), leading to translational repression or mRNA degradation (1–3). Given their fundamental roles in regulating gene expression, miRNAs have been implicated in a vast array of biological processes, including development, differentiation, and cellular homeostasis. Dysregulation of miRNA expression is frequently observed under various pathological conditions, especially cancer. Such dysregulation can stem from genomic alterations affecting miRNA loci (amplification, deletion, or translocation), aberrant activity of transcription factors controlling miRNA expression (e.g., c-Myc and p53), or defects in the miRNA biogenesis machinery (4). In the context of muscle biology, miRNAs play crucial roles in the histogenesis, development, growth, and regeneration of skeletal, cardiac, and smooth muscle across multiple species (5–9). A subset of miRNAs, often termed “MyomiRs” (e.g., miR-1, miR-133, and miR-206), are highly enriched in muscle tissues and are essential regulators of myogenesis—the process by which committed myoblasts proliferate, differentiate, and fuse to form multinucleated myotubes, the precursors of mature muscle fibers (10–13). This complex process involves tightly controlled temporal and spatial expression of muscle-specific genes, orchestrated by key myogenic regulatory factors, such as MyoD and Myogenin (MyoG) (14–16). Specific miRNAs modulate these pathways; for example, miR-26a inhibits myogenic differentiation by targeting Smad1 and Smad4 in the TGF-*β*/BMP signaling pathway (17–19).

miR-182-5p is a highly conserved miRNA that is implicated in diverse biological functions, including immune response, DNA repair, liver regeneration, and cancer progression. Its expression has also been reported to change in specific cell types under certain stimuli, such as in airway smooth muscle cells upon TNF-*α* stimulation (20). Notably, miR-182 has been established as a critical metabolic regulator that improves glucose metabolism by modulating FoxO1/PDK4 levels in muscle tissue (21). Separately, miR-182-5p and miR-103a-3p were identified as pathogenic drivers in leiomyomatosis (22). Although the importance of miR-182-5p in various cellular contexts is established, its specific role and mechanism of action during skeletal muscle myogenesis, particularly in commonly used model systems, such as C2C12 myoblasts remain unknown. Therefore, in this study, we investigated the functional role of miR-182-5p in regulating the differentiation of C2C12 cells and aimed to identify the downstream target genes involved in this process.

## 2 Materials and methods

### 2.1 Cell culture

Skeletal C2C12 myoblasts (CRL-1772; ATCC) were removed from the liquid nitrogen tank and allowed to resuscitate. The cells were then cultured in a carbon dioxide incubator at 37°C with 5% CO_2_. For experiments, only those cells that had been passaged less than 30 times were used. The cells were exposed to a high-sugar medium containing 2% horse serum for induction of myogenic differentiation. The cell lines present in this study were obtained from American Type Culture Collection (ATCC).

### 2.2 Transfection

miR-182-5p mimic and miR-182-5p inhibitor were purchased from RiboBio (Guangzhou, China). The C2C12 cells were cultured in 6-well culture plates and grown to approximately 50–60% confluence, after which they were transfected with miR-182-5p mimic or miR-182-5p inhibitor using the riboFECTTMCP reagent (Ribobio, GuangZhou), following the manufacturer’s instructions.

### 2.3 Giemsa staining

Cells were transfected for 24 h and then the transfection mixture was replaced with a myogenic differentiation medium to induce myogenic differentiation on days 2, 4, 6, and 8. The degree of myogenic differentiation of Giemsa-stained C2C12 cells was observed under a microscope according to the manufacturer’s instructions. As described below, the cells were washed with phosphate-buffered saline (PBS), treated with 500 μL of anhydrous methanol for 10 min, and then stained with 400 μL of Giemsa dye for 1 h. Subsequently, the stained cells were washed with distilled water to remove the excess dye, observed under a microscope, and photographed.

### 2.4 Immunofluorescence assay

Cells were seeded into 4-well plates and washed with PBS. The assay was performed according to the manufacturer’s instructions. The cells were fixed with 200 μL of cell tissue fixative for 15 min, permeabilized with 200 μL of 0.5% TritonX-100 for 10 min, blocked with 200 μL of 1% bovine serum albumin for 30 min, and then incubated overnight with 100 μL of primary antibody at 4°C. Thereafter, these cells were incubated with 100 μL of secondary antibody for 1 h and stained with DAPI. Before incubation with each new reagent, the cells were washed three times with 200 μL PBS for 5 min each time. The stained cells were with antifluorescence quenching sealer and observed under a confocal microscope.

### 2.5 Quantitative real-time PCR (qRT-PCR)

Total RNA, including miRNAs, was extracted from C2C12 cells using the PrimeScript RT reagent Kit with gDNA Eraser kit, following the manufacturer’s instructions. The concentration of total RNA was measured using a microspectrophotometer, and 100 ng RNA was reverse-transcribed to cDNA using the Thermal Sciences First Strand cDNA Synthesis Kit. The cells were induced in 6-well cell culture plates, and the myogenic differentiation medium was discarded. The cells were collected in trypsin-free tubes via trypsin digestion. RNA was extracted on ice in an enzyme-free environment. The PrimeScript RT reagent Kit with gDNA Eraser (Perfect Real Time) was used for miRNA reverse transcription. MicroRNA reverse transcription primers were synthesized by Rebo. Real-time PCR of the marker gene and miR-182-5p was performed using TB Green Premix Ex Taq II (Tli RNase H Plus). The primers used in the qRT-PCR experiments were synthesized by BGI.

### 2.6 Western blotting

Proteins were extracted using a Mammalian Protein Extraction Kit. The Mammalian Protein Extraction Reagent was cooled in advance of protein extraction. The bicinchoninic acid assay was used to determine the protein concentration for sample loading. Protein samples separated via SDS-PAGE were transferred onto nitrocellulose membranes using the wet transfer method. The membranes were then blocked by incubating them in 5% skim milk powder at 37°C for 1 h on a shaker. The following primary antibodies were used: Anti-GAPDH (Ab9485, Abcam), Anti-MYOD (PA5-23078, Thermo Fisher), and Anti-*α*-Tublin (11224-1-AP, Proteintech). Heavy chain (Ab11083, Abcam), primary antibodies were specifically conjugated to the target protein and incubated overnight at 4°C. The membranes were then rinsed three times with TBST and incubated with horseradish peroxidase-conjugated AffiniPure Goat Anti-Rabbit lgG (H + L) (SA00001-2, Proteintech) for 1 h at room temperature. The blots were developed using Pierce™ ECL Western Blotting Substrate and visualized with TANon-5200. The ImageJ software (NIH, USA) was used for densitometric analysis to determine the protein expression.

### 2.7 Dual-luciferase® reporter assay

A dual-luciferase reporter vector (E1960; Promega) containing target gene-binding site (s) was constructed. This vector was subsequently transfected into 293 T cells using Lipofectamine^®^ 2000 Reagent via liposome-mediated transfection. The luciferase activity was measured using a Dual-Luciferase Reporter Assay System. Firefly/Renilla ratios were normalized to the MUT + NC group (set as 100%).

### 2.8 Statistical analysis

Statistical analyses were performed using GraphPad Prism 9.5 (GraphPad Software). Between two groups, differences were assessed by unpaired two-tailed Student’s t-test. For multi-group comparisons, one-way ANOVA with Tukey’s *post hoc* test was applied. Data represent mean ± SEM of three biologically independent experiments. Significance levels: *p < 0.05, **p < 0.01, ***p < 0.001, ****p < 0.0001.

## 3 Results

### 3.1 miR-182-5p overexpression enhances myogenic differentiation in C2C12 cells

**Figure 1:**
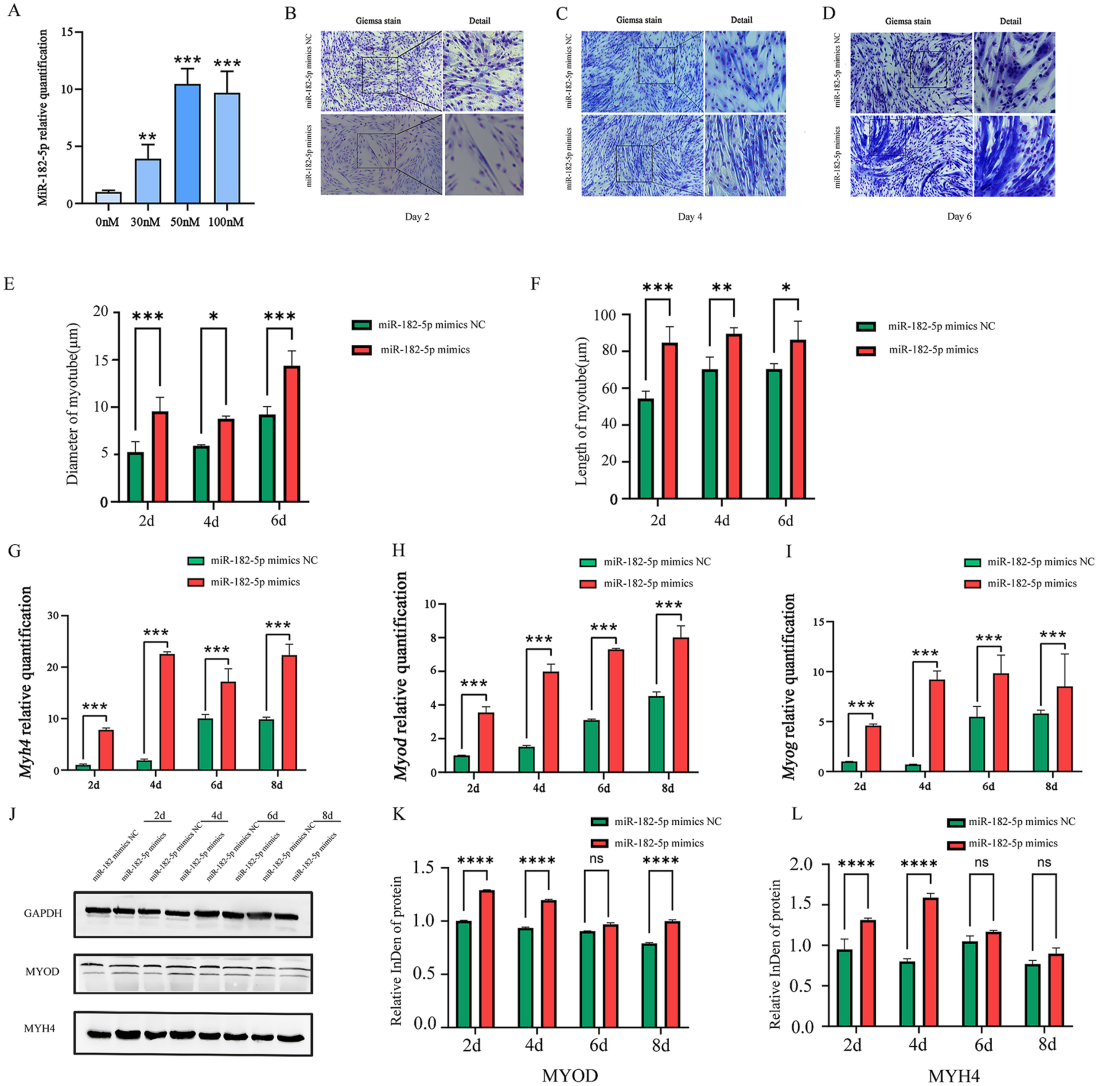
**(A)** Relative expression of miR-182-5p in C2C12 cells transfected with the miR-182-5p mimic at different concentrations. Compared with the group without miR-182-5p mimic addition, at 50 nM, the miR-182-5p mimic significantly enhanced the miR-182-5p expression. Data are presented as mean ± SEM of three independent experiments. **p < 0.01, ***p < 0.001 (Student’s *t*-test). **(B–D)** Giemsa staining of C2C12 cells transfected with the miR-182-5p mimic during differentiation (days 2, 4, and 6). **(E,F)** Quantitative analysis confirmed myotubes with significantly greater length and larger diameter in the miR-182-5p mimic group. Data are presented as mean ± SEM of three independent experiments. *p < 0.05, **p < 0.01, ***p < 0.001 (Student’s *t*-test). **(G–I)** mRNA levels of *Myh4*, *Myod*, and *Myog* were significantly upregulated in mimic-transfected cells. Data are presented as mean ± SEM of three independent experiments. ***p < 0.001 (Student’s *t*-test). **(J–L)** Western blot analysis confirmed the increased in MYOD and MYH4 protein levels. Data are presented as mean ± SEM of three independent experiments. ****p < 0.0001 (Student’s *t*-test).

**Figure 2:**
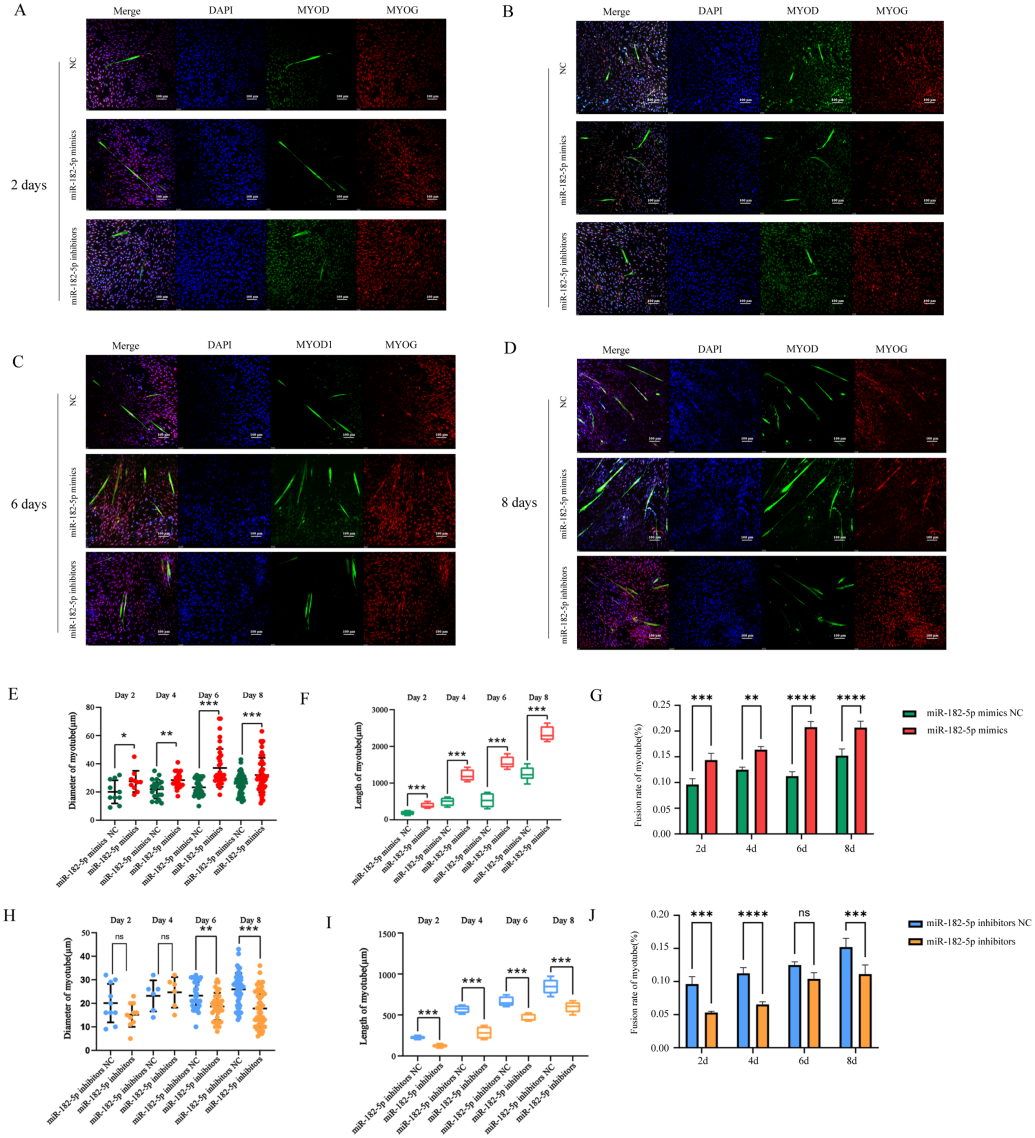
**(A–D)** Immunofluorescence staining (MYOD, green) showed greater length and larger diameter myotubes in mimic-transfected cells compared with those in controls and showed reduced myotube formation in the inhibitor group (days 2–8). Nuclei were counterstained with DAPI (blue). Fusion rate of myotubes were calculated based on the number of MHC-stained cells. **(E–G)** Quantitative analysis of immunofluorescence images revealed that the myotube length, diameter, and fusion rate were significantly increased in the miR-182-5p mimics group compared to the miR-182-5p mimics NC group. **(H–J)** Quantitative analysis of immunofluorescence images demonstrated that the miR-182-5p inhibitor group exhibited significantly decreased myotube length, diameter, and fusion rate compared to the miR-182-5p mimics negative control (NC) group. Data are presented as mean ± SEM of three independent experiments. *p < 0.05, **p < 0.01, ***p < 0.001 (Student’s *t*-test).

Prior to transfection experiments, we systematically determined the optimal concentration of the miR-182-5p mimic via dose–response screening. Quantitative analysis revealed that transfection with 50 nM miR-182-5p mimic resulted in significantly higher relative expression levels of miR-182-5p than achieved with other concentrations (*p* < 0.001), establishing this concentration as optimal for subsequent C2C12 cell experiments (Figure 1A). To investigate the role of miR-182-5p in myogenic differentiation, we performed a time-course analysis using Giemsa staining. During days 2–6 of differentiation, the mimics-transfected cells exhibited greater length and larger diameter myotubes than the controls (Figures 1B–F). Molecular analysis revealed a significant upregulation of myogenic differentiation markers at both the transcriptional and translational levels. qRT-PCR showed elevated mRNA expression of *Myh4*, *Myod*, and *Myog* in transfected cells throughout the differentiation timeline (days 2–8; Figures 1G–I). Western blot analysis confirmed the corresponding increase in MYH4 and MYOD protein levels, with statistically significant differences maintained across all time points (Figures 1J–L). Immunofluorescence analysis revealed that miR-182-5p-transfected cells consistently developed longer and thicker myotubes compared with controls from days 2 to 8 of differentiation (Figure 2). These results established that miR-182-5p overexpression enhanced the myogenic differentiation of C2C12 cells.

### 3.2 miR-182-5p suppression attenuates myogenic differentiation in C2C12 cells

**Figure 3:**
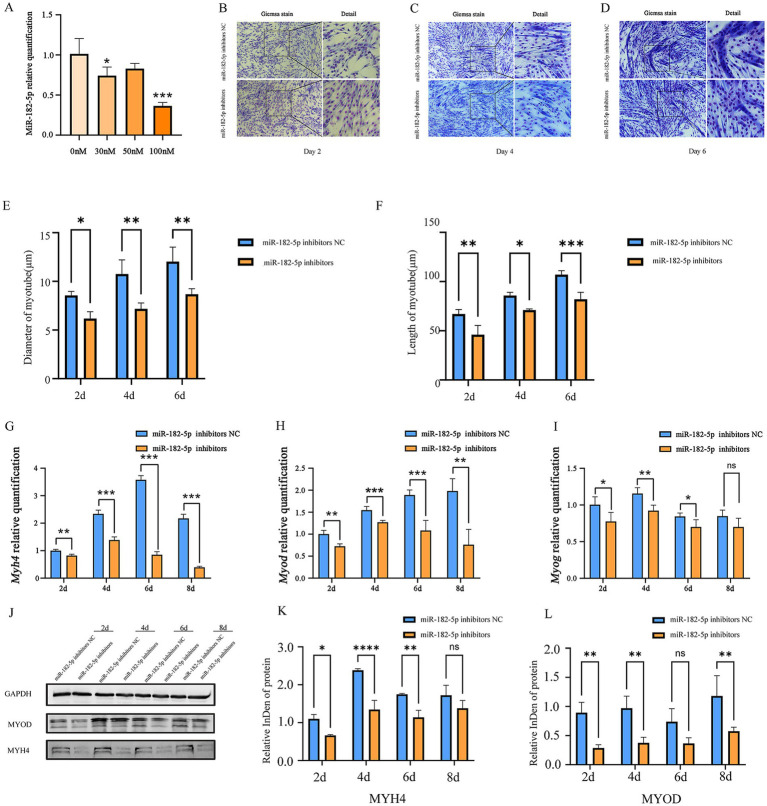
**(A)** Relative expression of miR-182-5p in C2C12 cells transfected with the miR-182-5p inhibitor at different concentrations. Compared with the group without miR-182-5p inhibitor addition, at 100 nM, the miR-182-5p inhibitor significantly inhibited the miR-182-5p expression Data are presented as mean ± SEM of three independent experiments. *p < 0.05, ***p < 0.001 (Student’s *t*-test). **(B–D)** Giemsa staining revealed shorter and smaller diameter myotubes in inhibitor-transfected C2C12 cells (days 2, 4, and 6). **(E,F)** Myotube length and diameter were significantly reduced in the miR-182-5p inhibitor-treated group compared to controls. Data are presented as mean ± SEM of three independent experiments. *p < 0.05, **p < 0.01, ***p < 0.001 (Student’s *t*-test). **(G–I)** mRNA levels of *Myh4*, *Myod*, and *Myog* were downregulated in the inhibitor group. Data are presented as mean ± SEM of three independent experiments. *p < 0.05, **p < 0.01, ***p < 0.001 (Student’s *t*-test). **(J–L)** Protein expression of MYOD and MYH4 was reduced in the inhibitor group. Data are presented as mean ± SEM of three independent experiments. *p < 0.05, **p < 0.01, ***p < 0.001, ****p < 0.0001(Student’s *t*-test).

We further identified 100 nM as the optimal miR-182-5p inhibitor concentration that was most effective in suppressing the expression of miR-182-5p in C2C12 cells (Figure 3A). While no significant morphological changes were observed on day 2 post-transfection, the inhibitor-transfected cells progressed to thinner myotubes than the controls by day 4 and 6 (Figures 3B–D). Quantitative morphometric analysis using the ImageJ software further revealed length and diameter were significantly reduced in miR-182-5p-suppressed cells from days 2 to 6 of differentiation (Figures 3E,F). Molecular characterization revealed the coordinated downregulation of myogenic markers across multiple layers of regulation. mRNA levels of *Myh4*, *Myod*, and *Myog* in inhibitor-transfected cells were significantly reduced throughout the differentiation period (days 2–8; Figures 3G–I). Correspondingly, western blot analysis confirmed marked reduction in MYH4 and MYOD protein expression, consistent with the observed alteration in the morphology of cells (Figures 3J–L). This phenotypic impairment was corroborated by immunofluorescence analysis, which revealed reduced myotube length and diminished diameter in the inhibitor-treated groups (Figure 2). Collectively, these findings demonstrated that miR-182-5p suppression attenuates the myogenic differentiation of C2C12 cells.

### 3.3 Zbtb7a is identified as a direct target of miR-182-5p and negatively regulated during myogenesis

**Figure 4:**
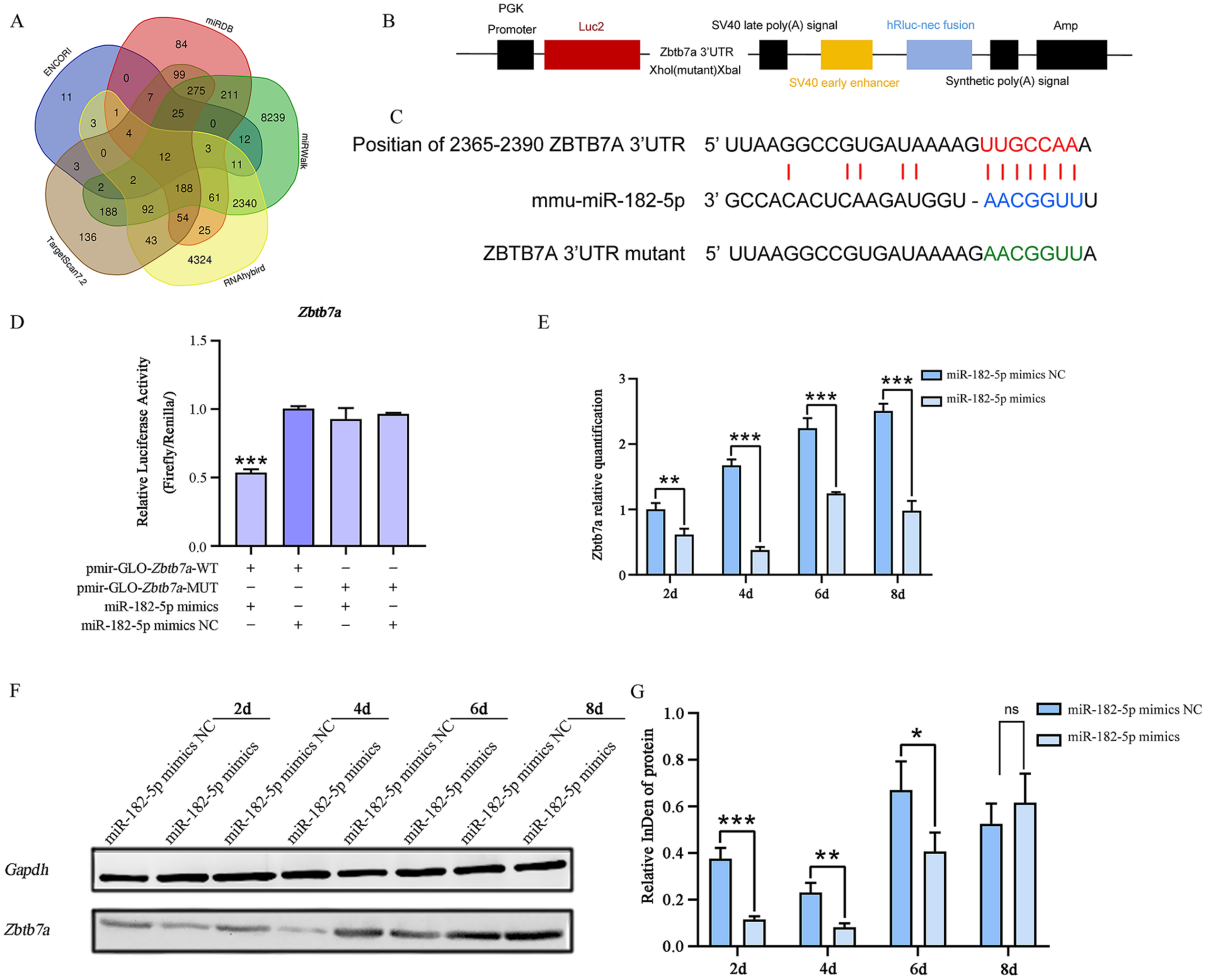
**(A)** Venn diagram of target genes for miRNA-182-5p predicted by multiple software programs. **(B,C)** Schematic of inserted Zbtb7a 3’-UTR and mutant 3’-UTR sequences. **(D)** Dual-luciferase assay in 293 T cells co-transfected with pmirGLO-Zbtb7a-WT and miR-182-5p mimic showed 50% reduction in Firefly/Renilla ratio vs. WT + NC controls. No suppression occurred with MUT constructs. NC: negative control mimic; MUT: binding site mutant. Confirming *Zbtb7a* as a target of miR-182-5p. Mutant (MUT) vectors showed no response. Data are presented as mean ± SEM of three independent experiments. Data are presented as mean ± SEM of three independent experiments. ***p < 0.001 (Student’s *t*-test). **(E)** qRT-PCR analysis: *Zbtb7a* mRNA was downregulated in mimic-transfected cells. Data are presented as mean ± SEM of three independent experiments. **p < 0.01, ***p < 0.001 (Student’s *t*-test). **(F,G)** Western blot analysis confirmed the inverse correlation between miR-182-5p and ZBTB7A protein levels. Data are presented as mean ± SEM of three independent experiments. *p < 0.05, **p < 0.01, ***p < 0.001 (Student’s *t*-test).

The candidate target genes predicted through multiple databases (ENCORI, miRDB, miRWalk, RNAhybrid, and TargetScan7.2) include: Arf4, Rere, Zbtb7a, Aebp2, Qk, Nuak1, Dab2ip, Casp2, Paip2, Tns3, Jmjd1c, 4930402H24Rik. Based on the binding probability scores of miR-182-5p target genes predicted by RNAhybrid, we selected Zbtb7a, Qk, Nuak1, Dab2ip, Casp2, and Tns3 for dual-luciferase assays. The results confirmed that Zbtb7a is a direct target of miR-182-5p. Cotransfection of cells with pmir-GLO-Zbtb7a-WT and miR-182-5p mimic resulted in a significant reduction in the Firefly/Renilla ratio, confirming the binding specificity (Figures 4A–D). To determine whether miR-182-5p regulates *Zbtb7a* expression during myogenesis, we evaluated *Zbtb7a* expression during the differentiation of C2C12 cells transfected with miR-182-5p mimic. Consistent with the identification of *Zbtb7a* as the direct target of miR-182-5p, qRT-PCR showed that the relative mRNA expression of *Zbtb7a* was significantly lower in cells transfected with miR-182-5p mimic than in the miR-182-5p mimic NC group (Figure 4E). These changes in *Zbtb7a* expression were reflected at the protein level, as determined using western blotting (Figures 4F,G), confirming that miR-182-5p negatively regulates *Zbtb7a* expression during the myogenic differentiation of C2C12.

### 3.4 Zbtb7a knockdown phenocopies miR-182-5p-mediated myogenic enhancement

**Figure 5:**
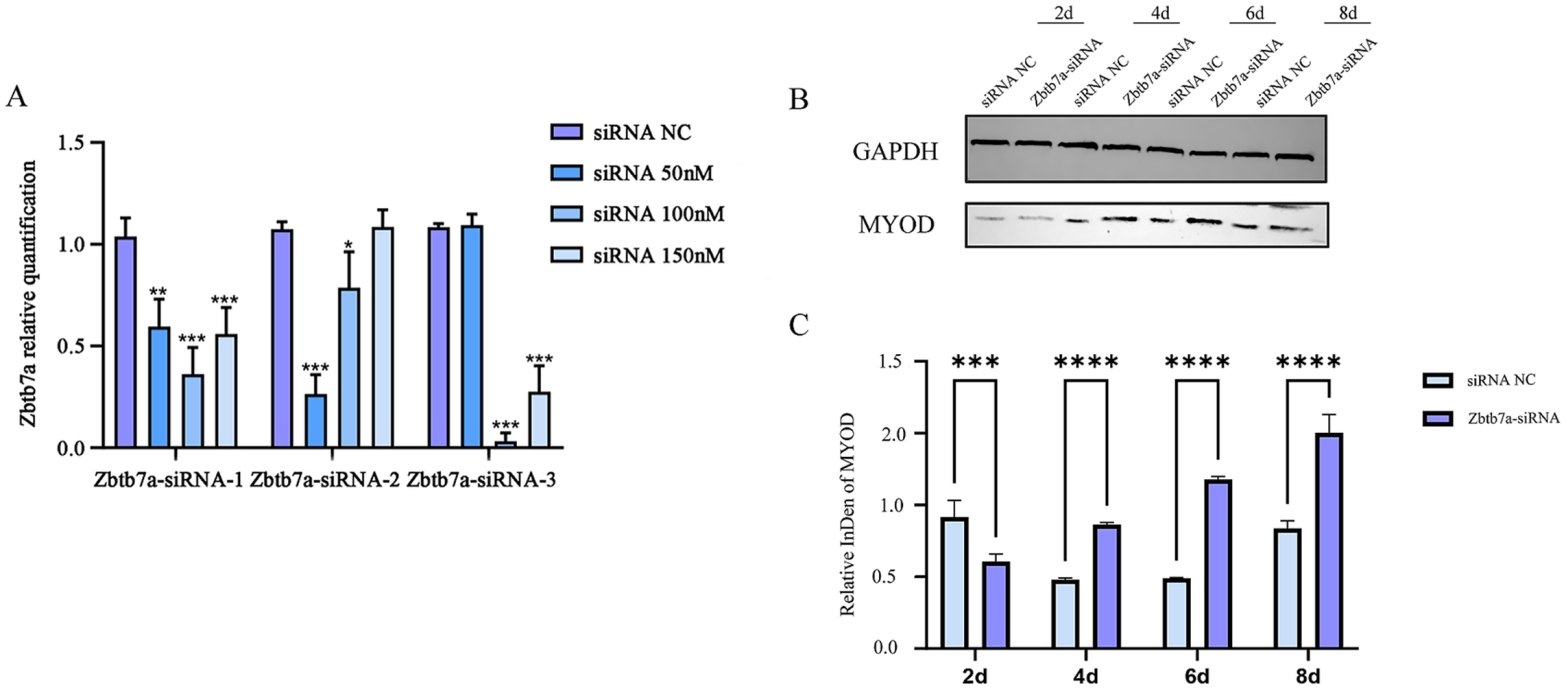
**(A)** Determination of the optimal transfection concentration of *Zbtb7a*-siRNAs. Data are presented as mean ± SEM of three biologically independent experiments. *p < 0.05, **p < 0.01, ***p < 0.001 (Tukey’s multiple comparisons test following one-way ANOVA). **(B,C)** After knockdown of the expression of *Zbtb7a* in C2C12 cells, mRNA and protein levels of myogenic differentiation marker genes were detected at days 2–8 of myogenic differentiation. Data are presented as mean ± SEM of three independent experiments. ***p < 0.001, ****p < 0.0001 (Student’s *t*-test).

To further identify *Zbtb7a* as a target gene for miRNA-182-mediated regulation of myogenic differentiation, its expression was knocked down using Zbtb7a-siRNA. Zbtb7a-siRNA-transfected C2C12 cells exhibited significantly increased induction of C2C12 myogenic differentiation compared with Zbtb7a-siRNA NC-transfected C2C12 cells (Figure 5).

## 4 Discussion

Skeletal myogenesis is a highly orchestrated process involving sequential activation of myogenic regulatory factors and downstream signaling pathways. The key factors, such as MYF5, MYOD, MYF6, and MYOG, drive the commitment and differentiation of myoblasts, which subsequently fuse to form multinucleated myotubes expressing structural proteins such as myosin heavy chains (e.g., MYH3 and MYH4). miRNAs are crucial regulators of this intricate network (23). For instance, the miR-183/96/182 cluster promotes the proliferation and differentiation of bovine myoblasts (24), and miR-378 promotes differentiation by targeting BMP4 (25). Building upon these findings, we investigated the specific role of miR-182-5p, a member of this cluster, in the differentiation of C2C12 murine myoblasts.

We found that miR-182-5p acts as a positive regulator of C2C12 myogenic differentiation. Overexpression of miR-182-5p, achieved via mimic transfection, significantly enhanced the differentiation process. This was evidenced by morphological changes (increased myotube formation) and was corroborated by the upregulation of key myogenic markers. In particular, we observed increased expression of MYOD, an early marker involved in myogenic commitment; MYOG, a crucial factor for terminal differentiation; and MYH4 (MHC IIb), a marker associated with mature, fused myotubes. These results suggest that miR-182-5p promotes the progression of differentiation at multiple stages. Conversely, inhibition of endogenous miR-182-5p impaired C2C12 differentiation, highlighting the necessity of this miRNA for a normal myogenic program. These results align with previous reports showing the promyogenic roles of the miR-183/96/182 cluster in bovine cells (24). The regulatory landscape of myogenesis involves numerous miRNAs, sometimes with opposing functions. For example, emerging evidence has highlighted the critical regulatory roles of miRNAs in myogenic processes. miR-182-5p was reported to negatively regulate myogenesis via Sesn2 suppression; Sesn2 knockout resulted in decreased myogenin (Myog) expression and elevated Pax7 levels, whereas its overexpression upregulated Myog and enhanced the predominance of slow-twitch myofiber (26). This regulatory axis suggests the dual functionality of miR-182-5p in modulating differentiation commitment and fiber-type specification. Complementing these findings, subsequent research revealed the essential involvement of miR-325-3p in actin cytoskeletal remodeling and differentiation dynamics of C2C12 myoblasts. Palmitic acid-mediated inhibition of myoblast differentiation was reported to be associated with upregulated miR-325-3p expression. Mechanistically, miR-325-3p directly targets the 3′-UTR of CFL2 mRNA— a critical mediator of myogenic differentiation—leading to its translational repression. Notably, transfection with a miR-325-3p mimic induced F-actin accumulation and facilitated nuclear translocation of YAP, creating a paradoxical scenario of enhanced proliferation coupled with compromised terminal differentiation. This novel miRNA-mediated regulatory mechanism involving CFL2 suppression and YAP activation expands our understanding of the epigenetic control of muscle development (27). These findings underscore the complexity of miRNA networks in myogenesis, revealing both convergent and divergent regulatory strategies. While miR-182-5p primarily modulates transcriptional regulators of differentiation, miR-325-3p apparently acts as an interface between cytoskeletal dynamics and mechanotransduction pathways. The contrasting outcomes of differentiation processes (enhancement vs. impairment) suggest context-dependent miRNA functionality, which warrants further investigation into the temporal expression patterns and pathway crosstalk. Our study contributes to this complexity by identifying *Zbtb7a* as a direct target of miR-182-5p during C2C12 differentiation. We confirmed this interaction using a dual-luciferase reporter assay, showing that miR-182-5p binds to the 3′-UTR of Zbtb7a to suppress its expression.

ZBTB7A (also known as LRF, FBI-1, and Pokemon) is a transcriptional repressor implicated in various cellular processes, including development, cell cycle control, and oncogenesis (28–30). Its known functions include repressing the tumor suppressor Rb and potentially impacting cell cycle exit, which is a prerequisite for terminal differentiation. It has also been implicated in metabolic regulation and prostate cancer progression via the modulation of transcription factor activity (31, 32). Although the precise downstream targets of ZBTB7A within the myogenic program remain to be fully elucidated, its established role as a transcriptional repressor suggests a plausible mechanism. By downregulating ZBTB7A, miR-182-5p may relieve the repression of genes essential for myogenic differentiation or cell cycle exit, thereby facilitating the overall process.

This study identifies miR-182-5p as a significant promyogenic factor in C2C12 cells, adding to a growing understanding of miRNA function in skeletal muscle development. We provide mechanistic insights by validating Zbtb7a as a direct target, suggesting that miR-182-5p promotes differentiation, at least in part, by alleviating ZBTB7A-mediated repression. However, we acknowledge that miR-182-5p likely targets other mRNAs, and that ZBTB7A itself regulates multiple downstream genes. Further research is warranted to comprehensively map the miR-182-5p-ZBTB7A axis and identify other interacting partners and downstream effectors within the complex network governing myogenesis. Investigating the precise mechanisms of ZBTB7A binding to its targets in myoblasts and exploring its potential epigenetic roles will also be worth investigating.

## 5 Conclusion

Our results establish a crucial role for miR-182-5p in promoting C2C12 myogenic differentiation by directly targeting and suppressing the transcriptional repressor Zbtb7a. These findings enhance our understanding of the molecular regulation of myogenesis, and highlight a potential pathway for the modulation of muscle development and regeneration.

## Data Availability

The raw data supporting the conclusions of this article will be made available by the authors, without undue reservation.
