# Supplementary figures and images for: miRNA-182-5p promotes myogenic differentiation of C2C12 cells via the suppression of ZBTB7A
Source: Front Vet Sci. 2025 Jul 30;12:1637277. doi: 10.3389/fvets.2025.1637277 (PMC12345297; doi:10.3389/fvets.2025.1637277)

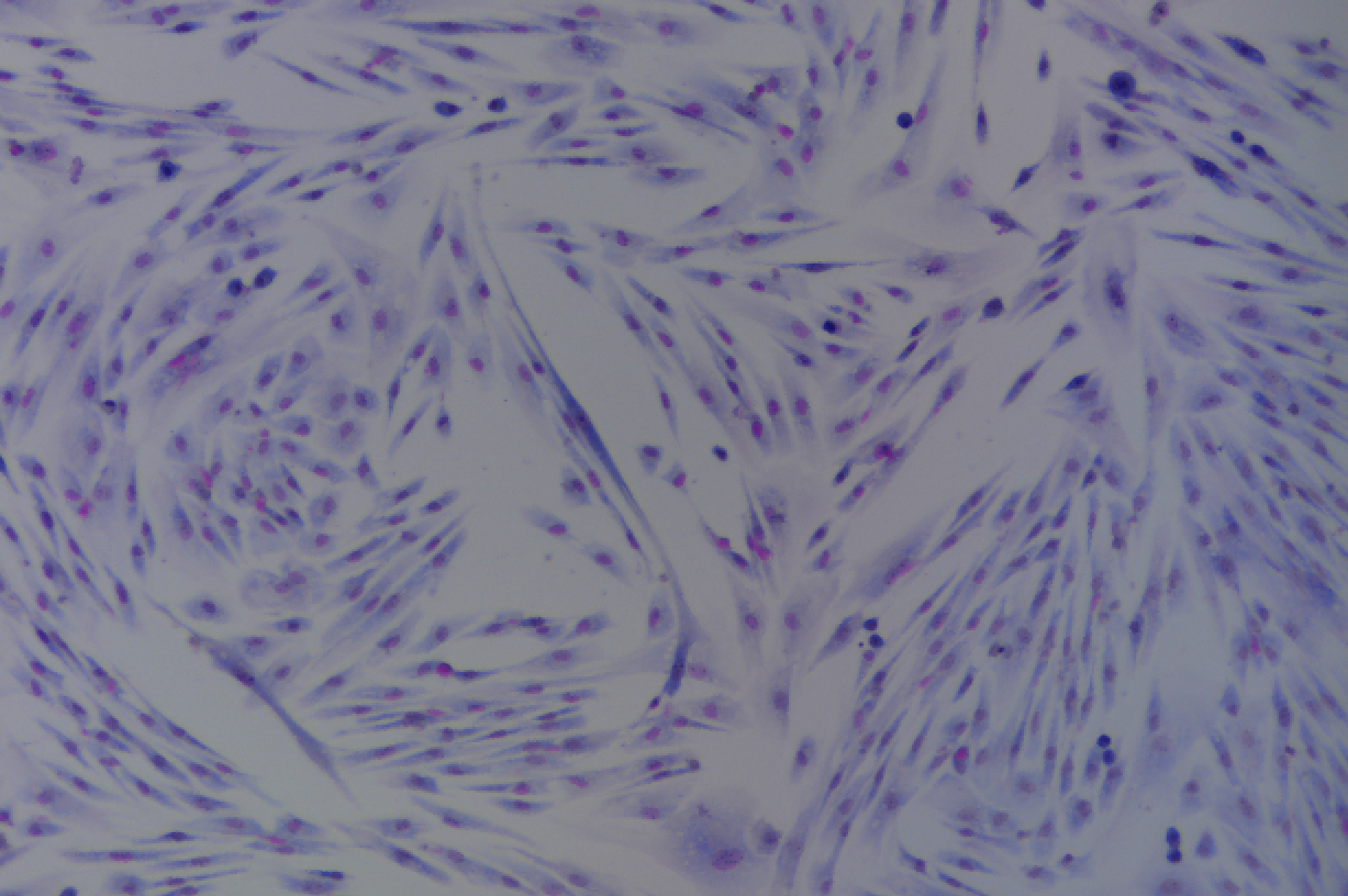

Supplement: Supplementary file 1 [file Data_Sheet_1.zip › Original Images/Giemsa/Fig1B(3) 2d mimics.png]

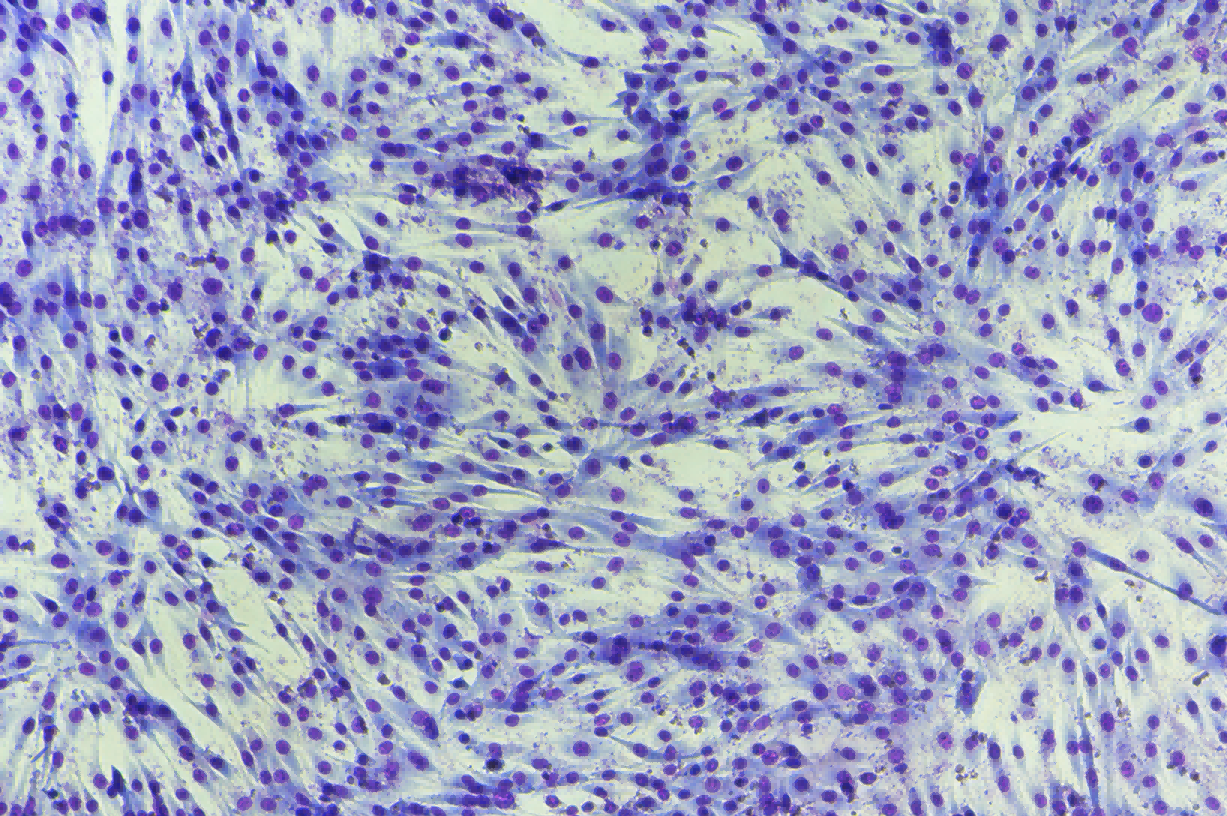

Supplement: Supplementary file 1 [file Data_Sheet_1.zip › Original Images/Giemsa/Fig1B(3) 2d NC.png]

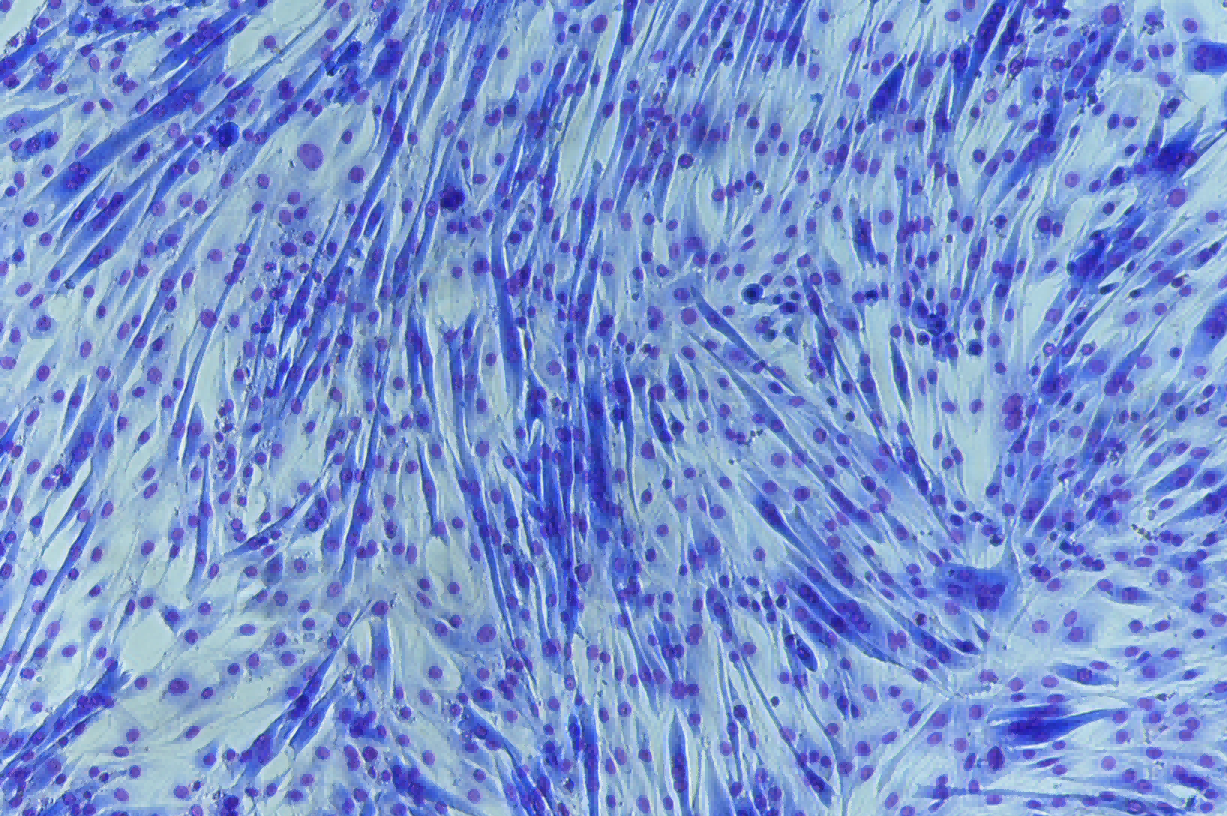

Supplement: Supplementary file 1 [file Data_Sheet_1.zip › Original Images/Giemsa/Fig1B(3) 4d mimics.png]

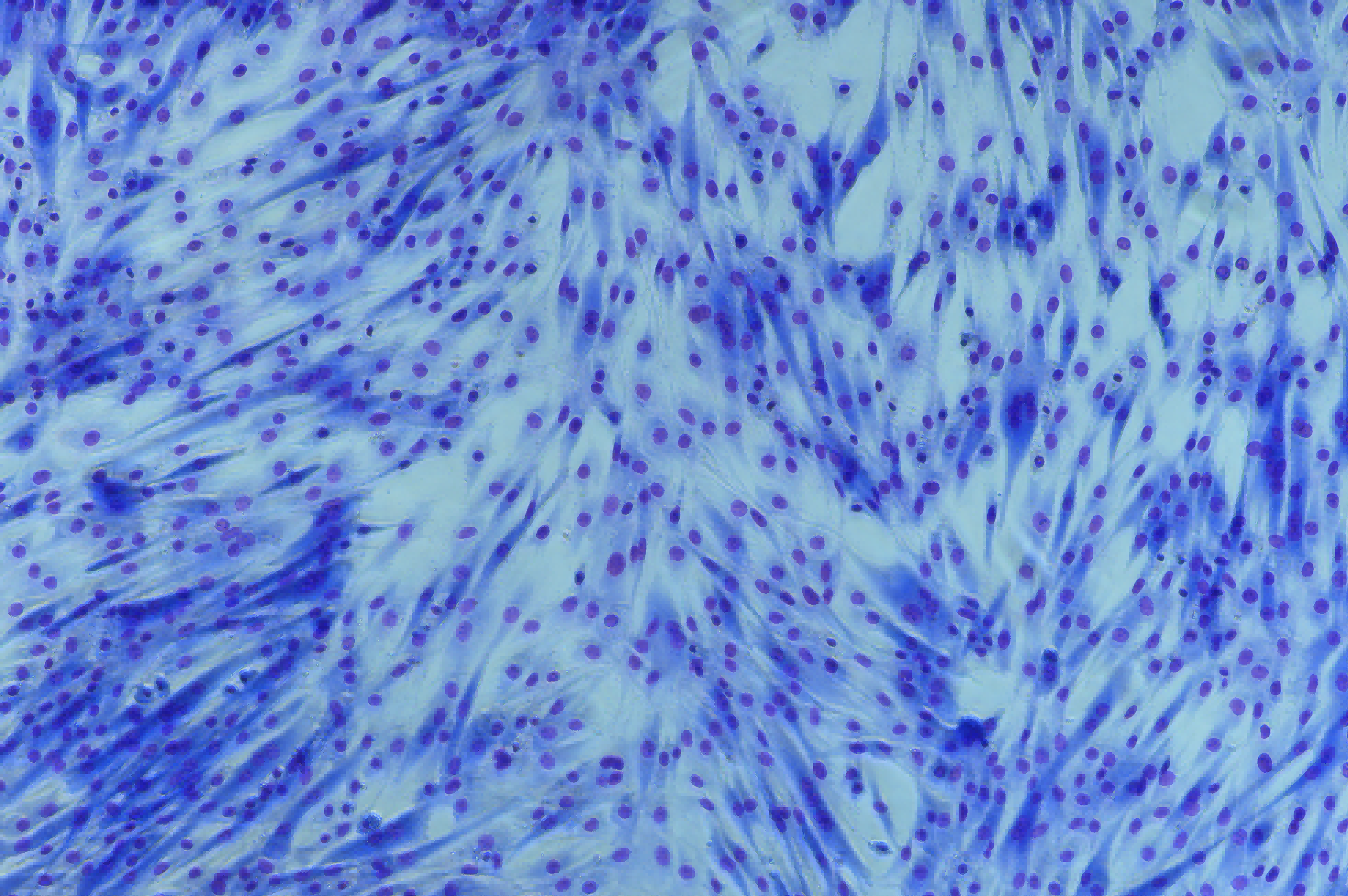

Supplement: Supplementary file 1 [file Data_Sheet_1.zip › Original Images/Giemsa/Fig1B(3) 4d NC.png]

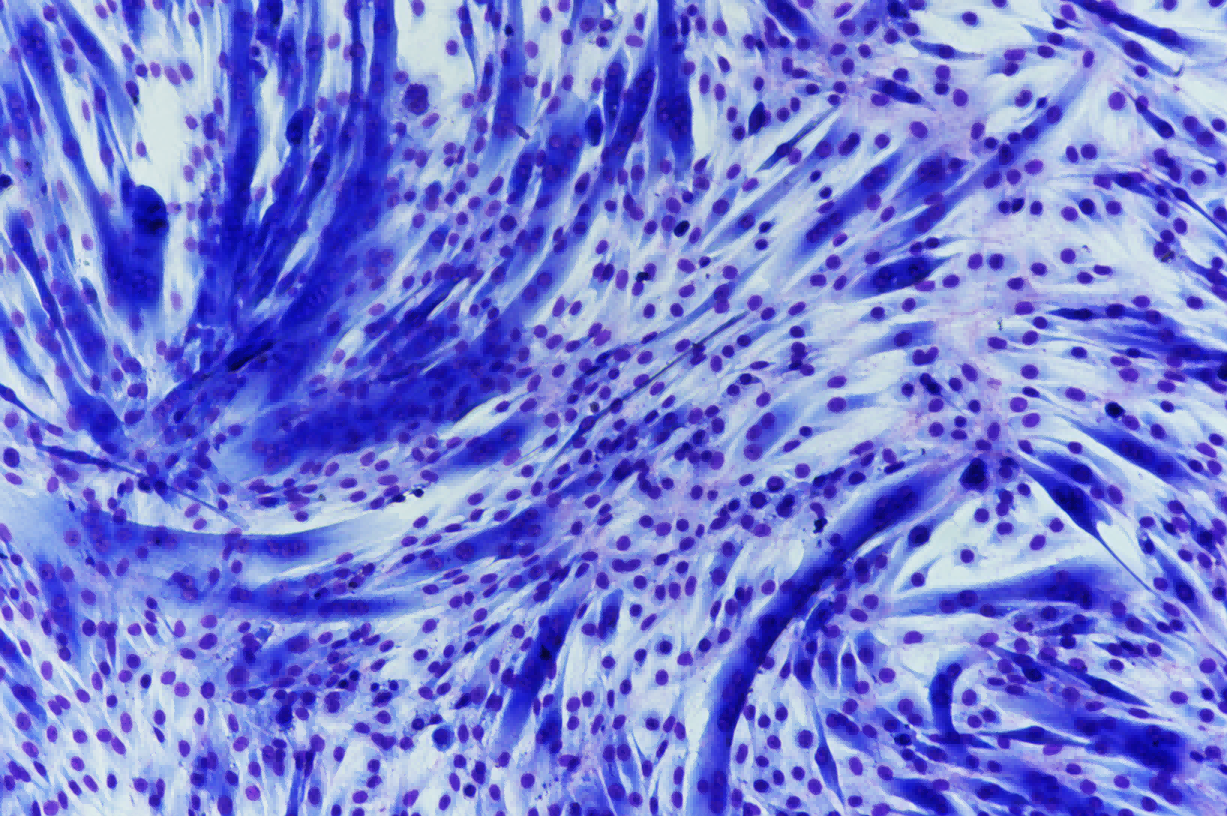

Supplement: Supplementary file 1 [file Data_Sheet_1.zip › Original Images/Giemsa/Fig1B(3) 6d mimics.png]

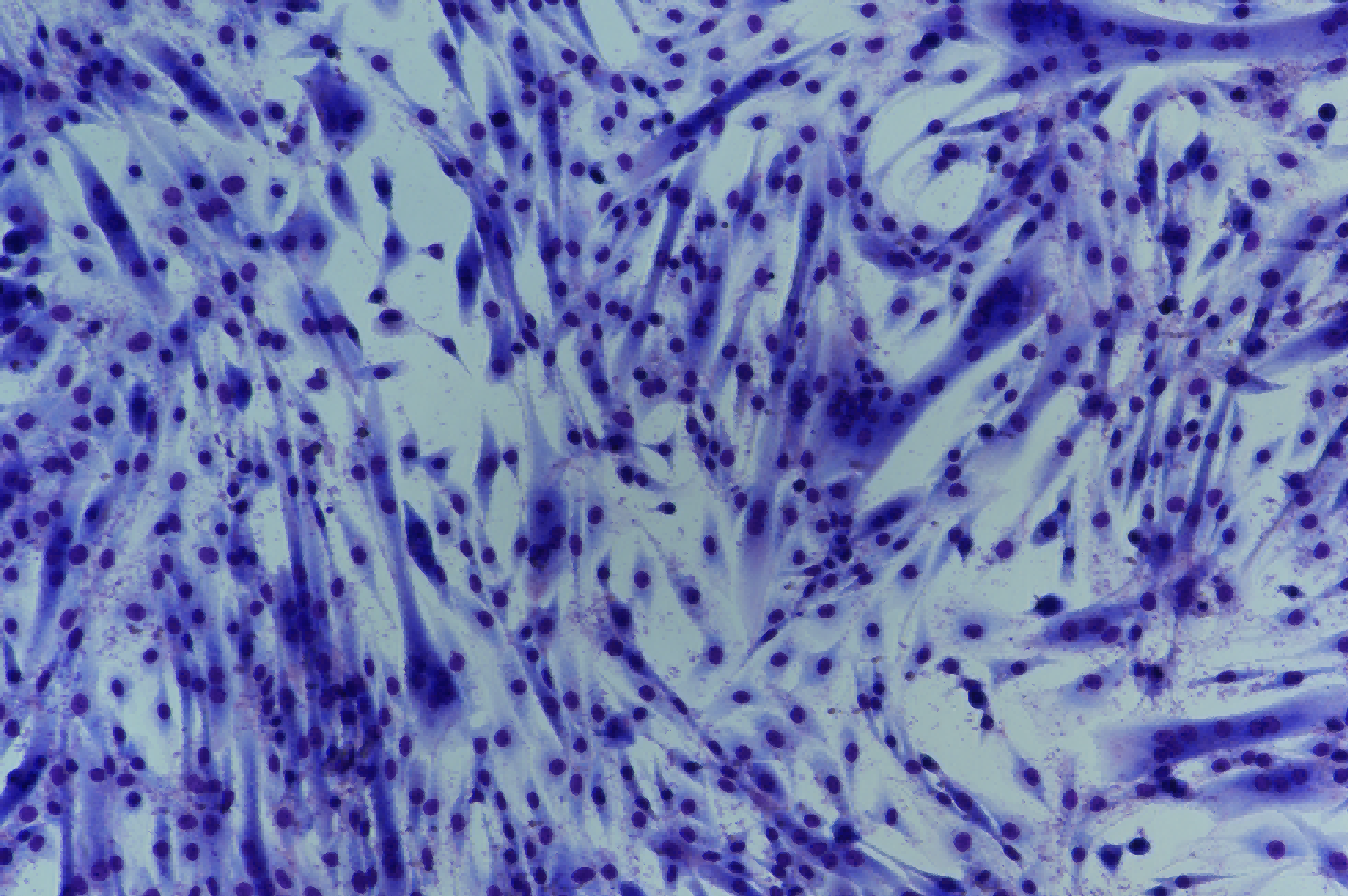

Supplement: Supplementary file 1 [file Data_Sheet_1.zip › Original Images/Giemsa/Fig1B(3) 6d NC.png]

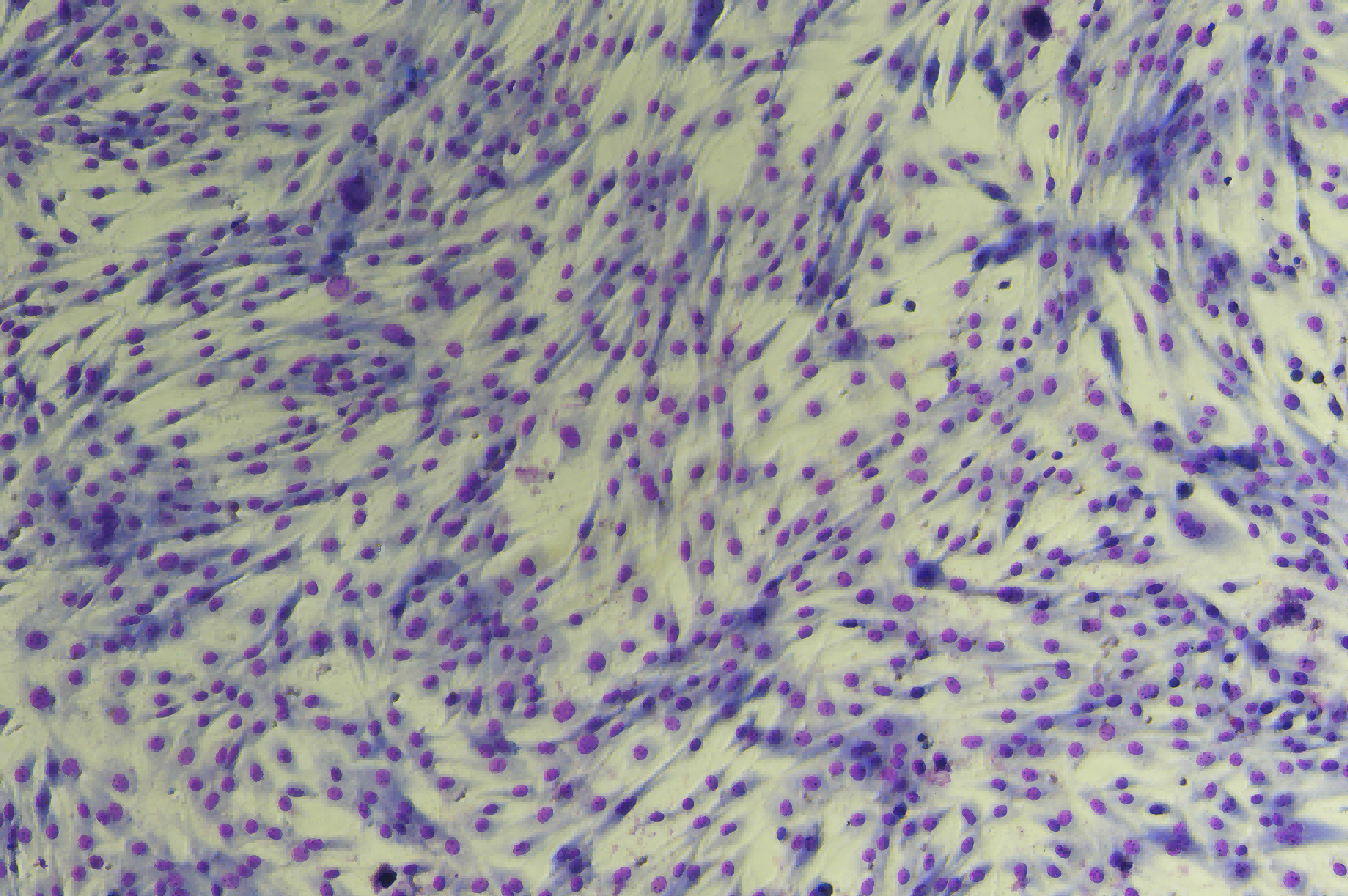

Supplement: Supplementary file 1 [file Data_Sheet_1.zip › Original Images/Giemsa/Fig2B(3) 2d inhibitor.png]

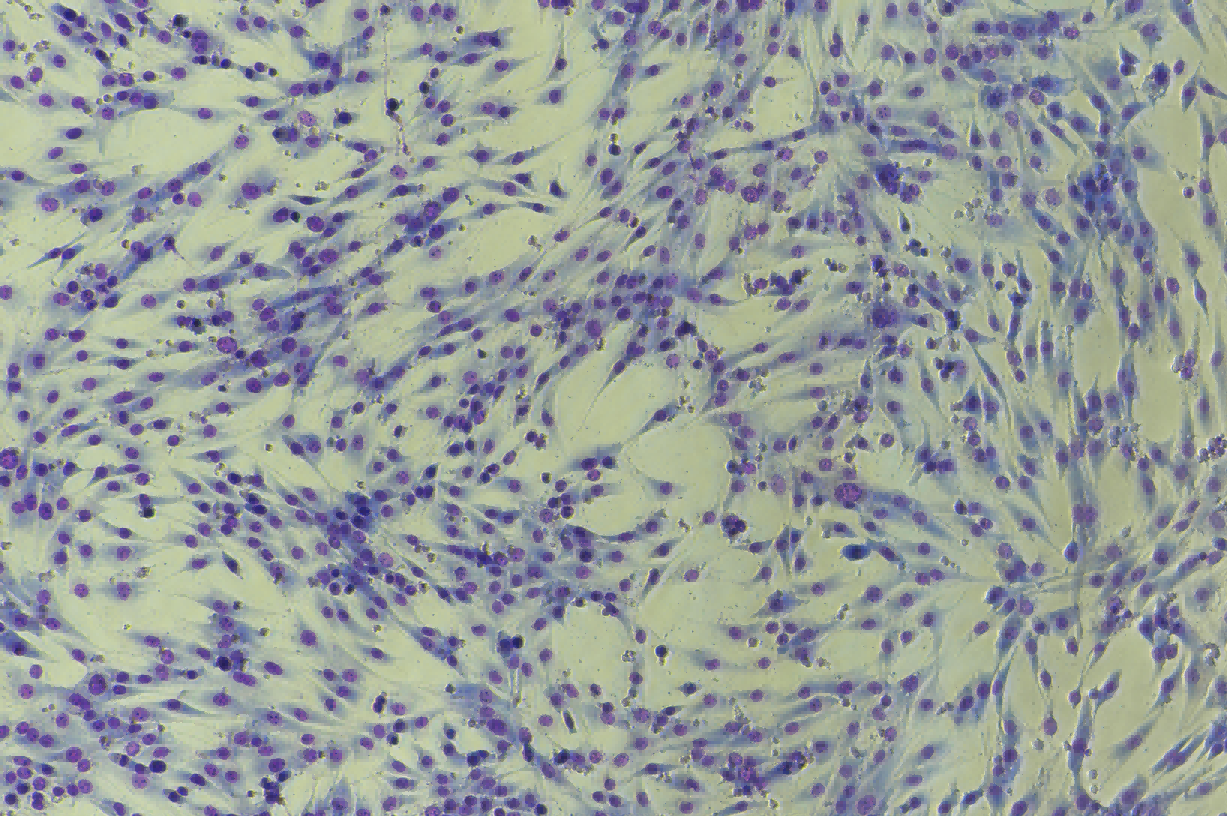

Supplement: Supplementary file 1 [file Data_Sheet_1.zip › Original Images/Giemsa/Fig2B(3) 2d NC.png]

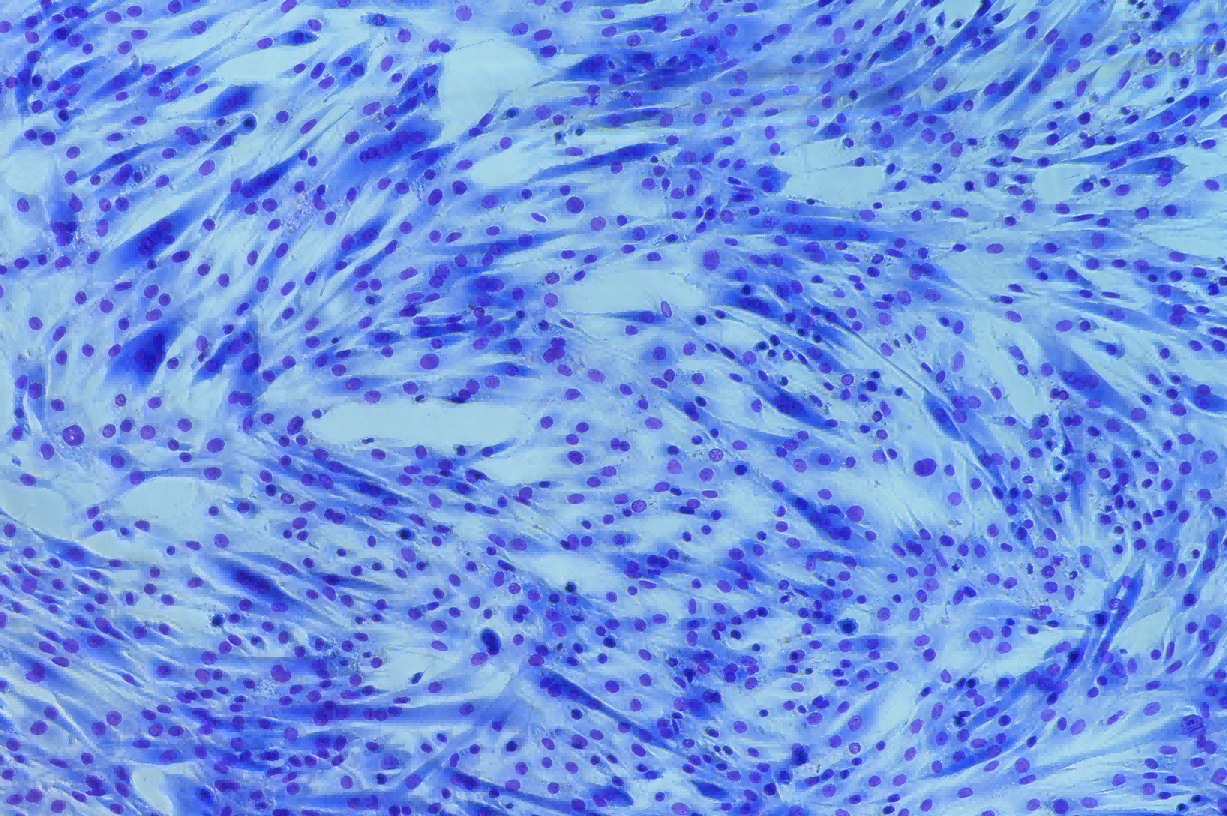

Supplement: Supplementary file 1 [file Data_Sheet_1.zip › Original Images/Giemsa/Fig2B(3) 4d inhibitor.png]

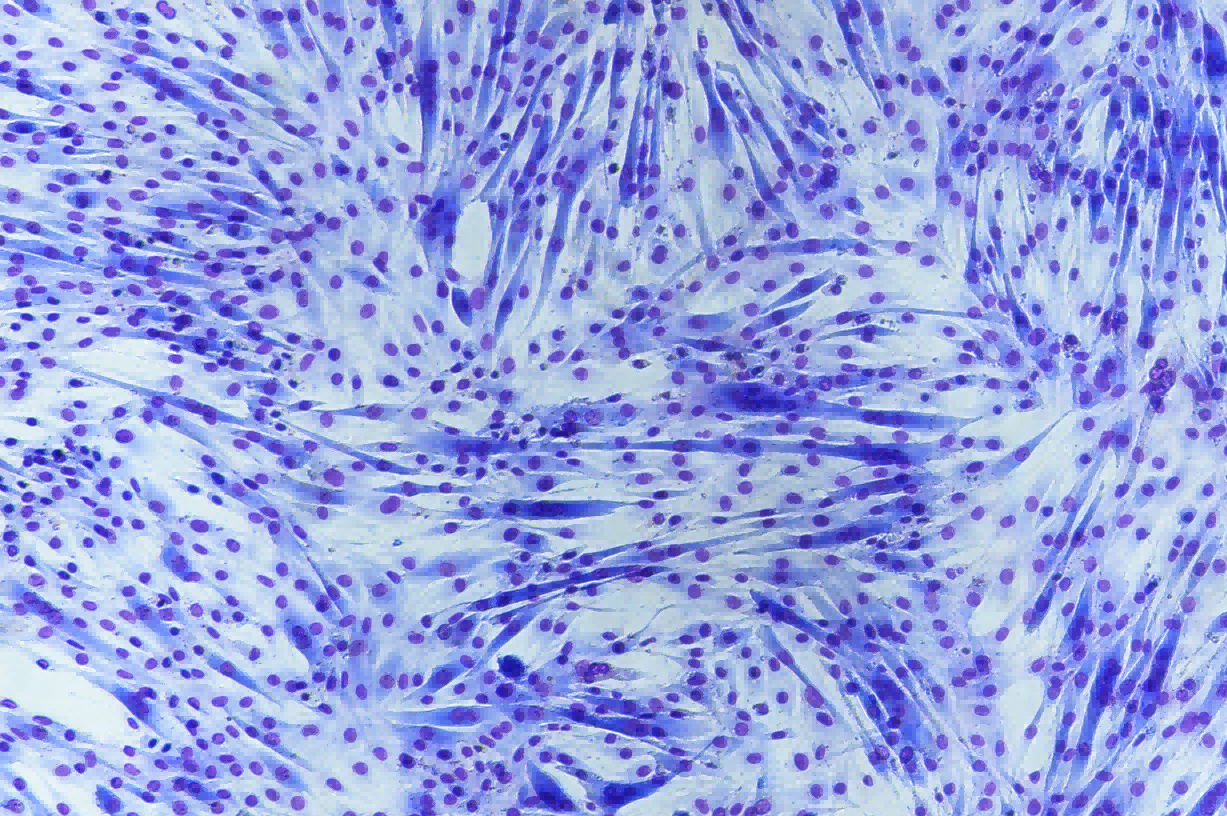

Supplement: Supplementary file 1 [file Data_Sheet_1.zip › Original Images/Giemsa/Fig2B(3) 4d NC.png]

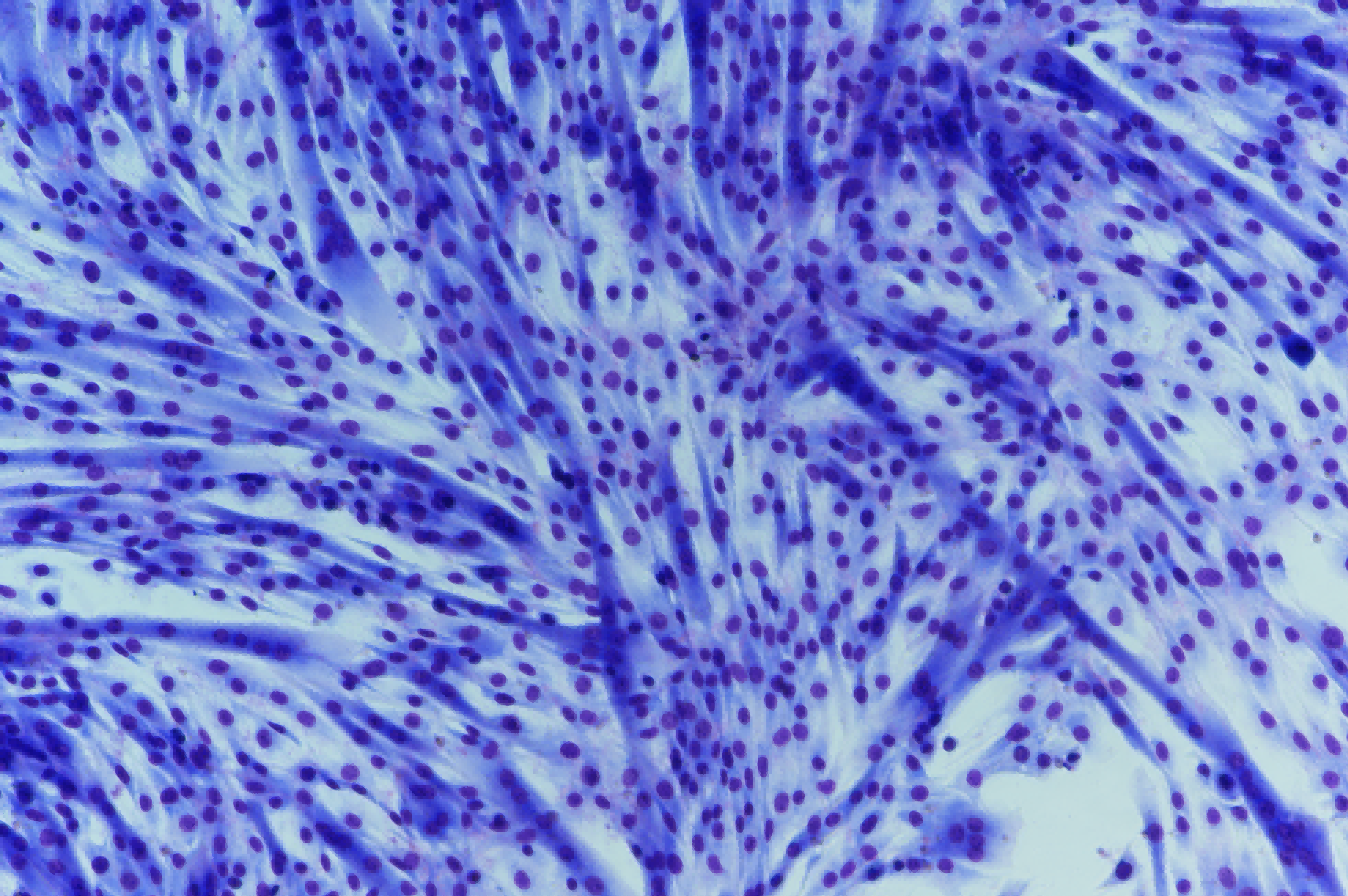

Supplement: Supplementary file 1 [file Data_Sheet_1.zip › Original Images/Giemsa/Fig2B(3) 6d inhibitor.png]

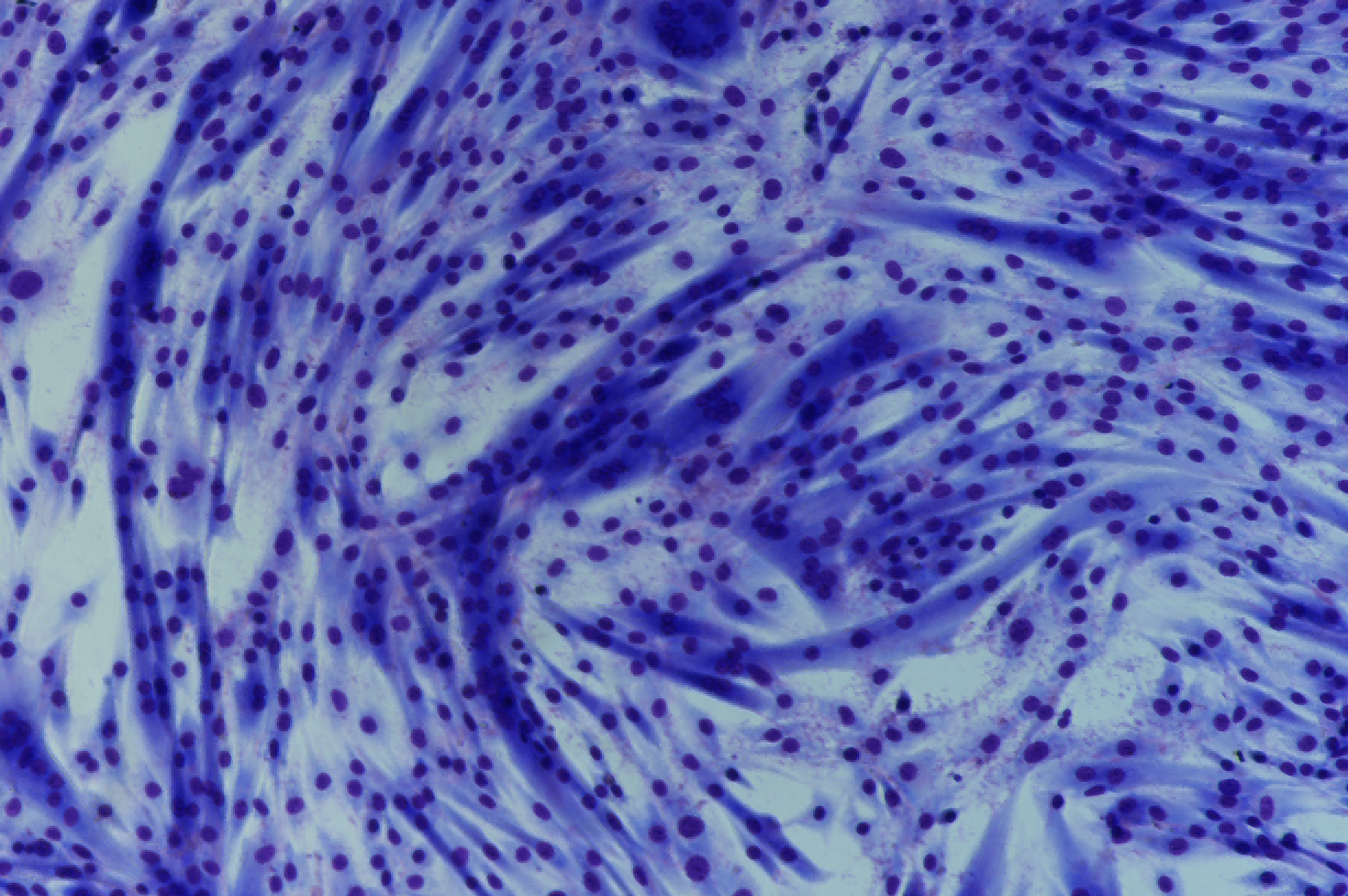

Supplement: Supplementary file 1 [file Data_Sheet_1.zip › Original Images/Giemsa/Fig2B(3) 6d NC.png]

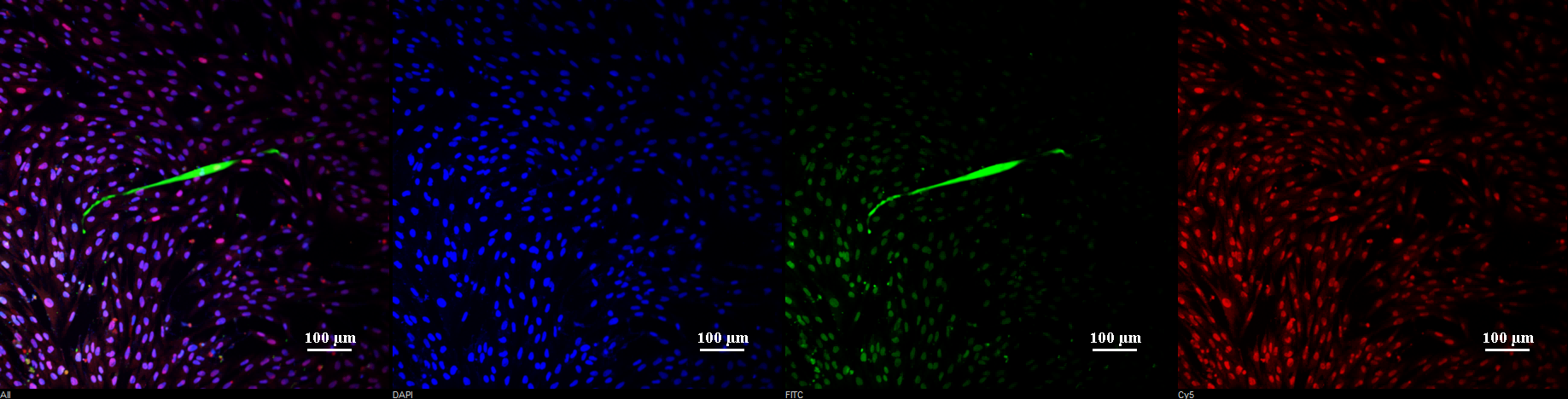

Supplement: Supplementary file 1 [file Data_Sheet_1.zip › Original Images/Immunofluorescence Staining/2d ctrl.png]

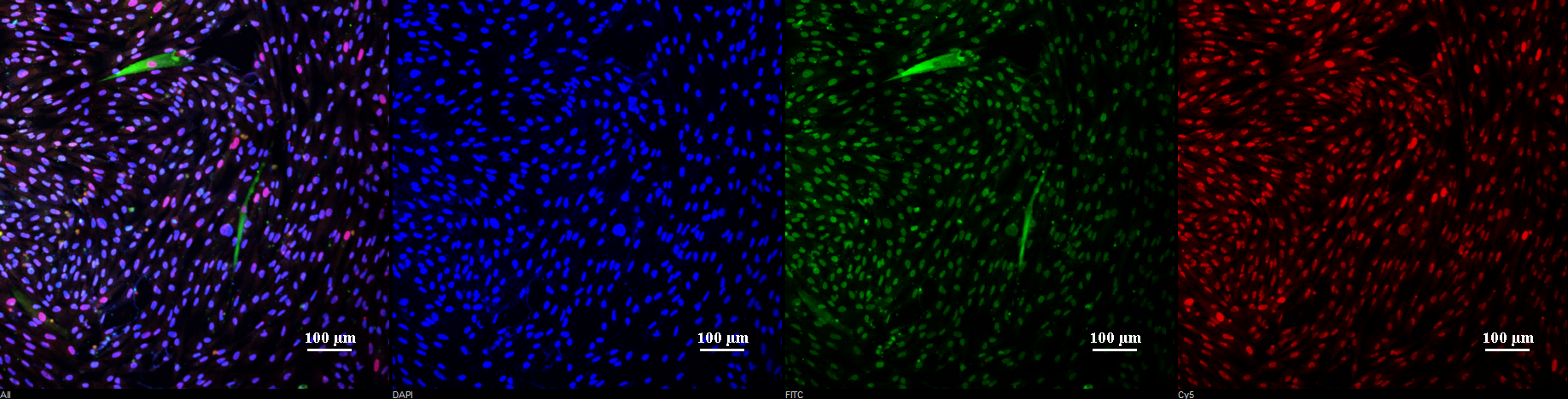

Supplement: Supplementary file 1 [file Data_Sheet_1.zip › Original Images/Immunofluorescence Staining/2d inhibitor.png]

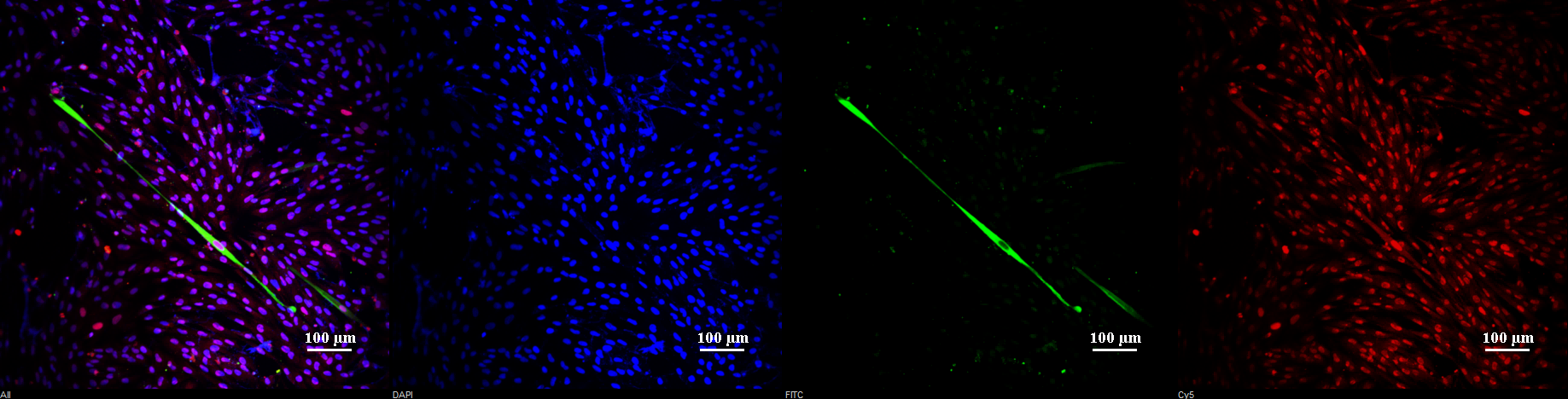

Supplement: Supplementary file 1 [file Data_Sheet_1.zip › Original Images/Immunofluorescence Staining/2d mimics.png]

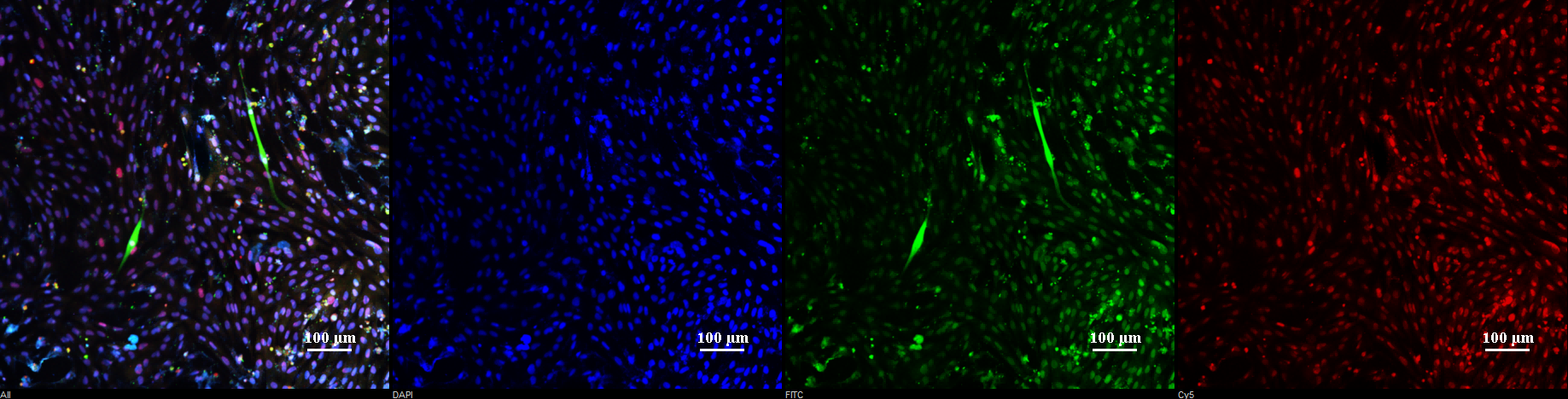

Supplement: Supplementary file 1 [file Data_Sheet_1.zip › Original Images/Immunofluorescence Staining/4d ctrl.png]

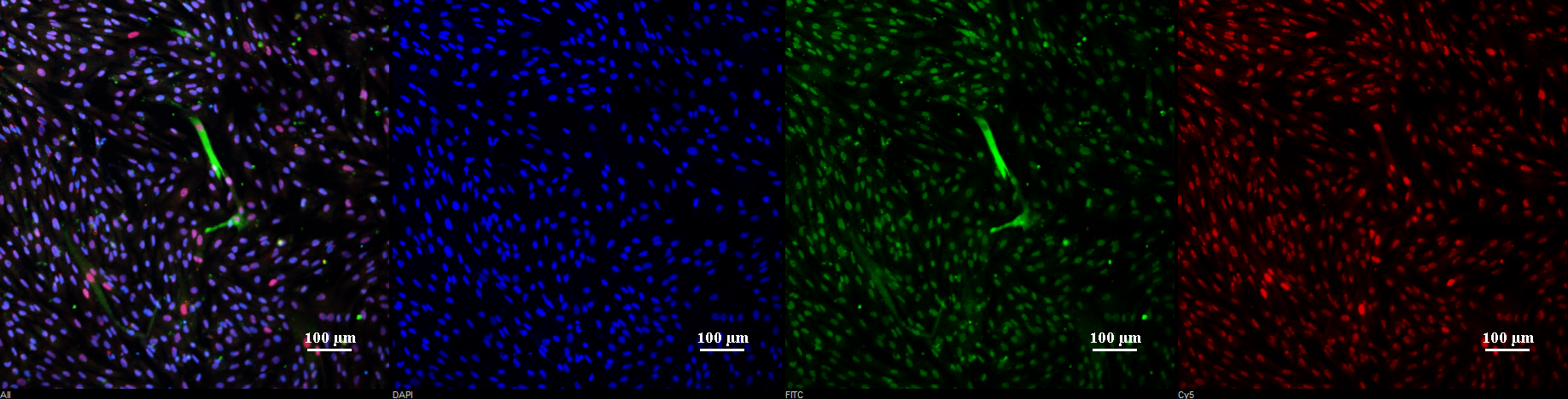

Supplement: Supplementary file 1 [file Data_Sheet_1.zip › Original Images/Immunofluorescence Staining/4d inhibitor.png]

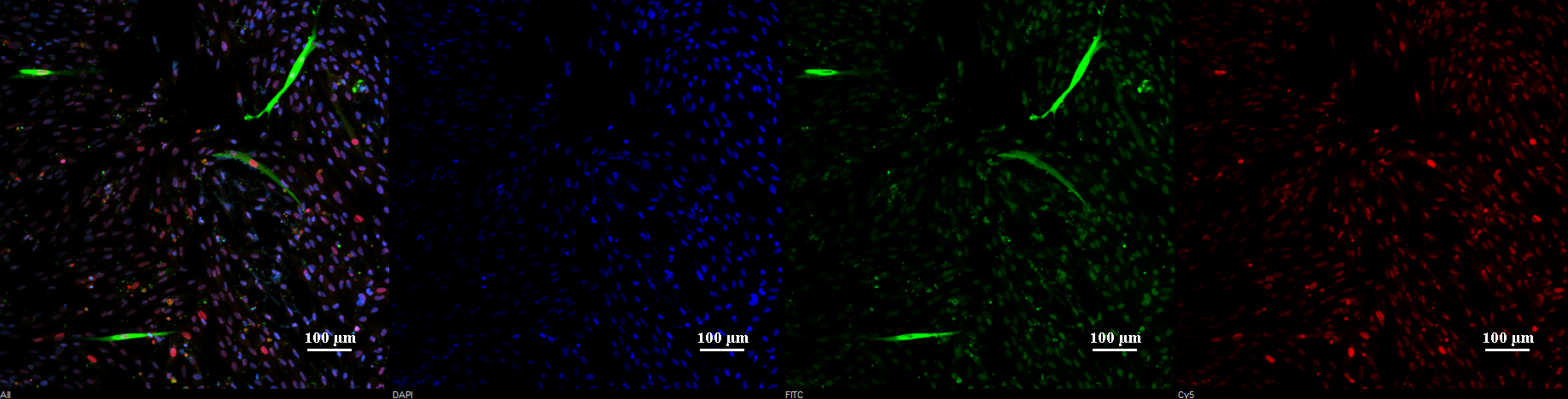

Supplement: Supplementary file 1 [file Data_Sheet_1.zip › Original Images/Immunofluorescence Staining/4d mimics.png]

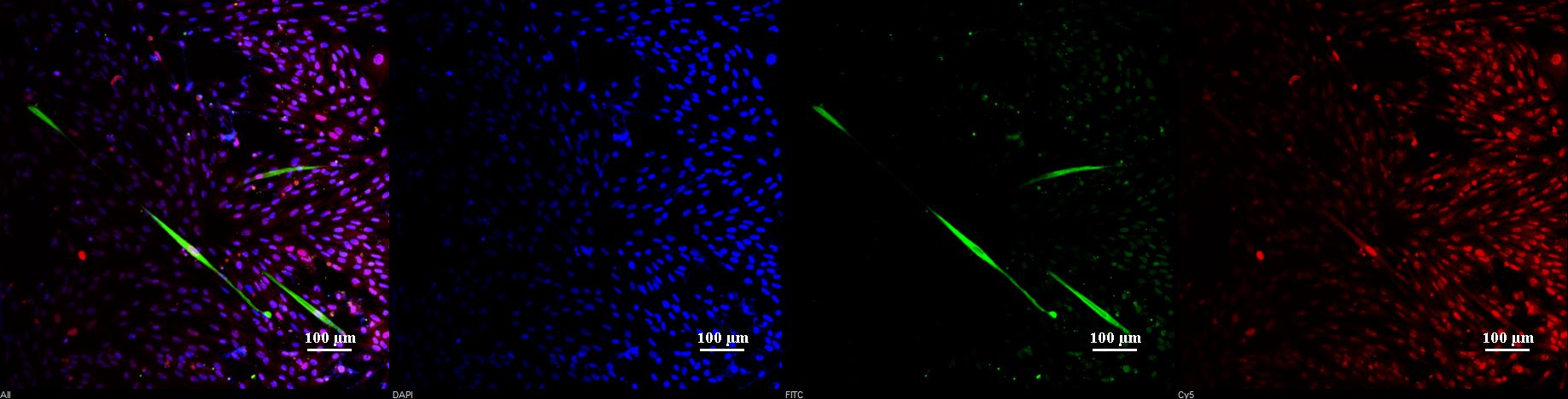

Supplement: Supplementary file 1 [file Data_Sheet_1.zip › Original Images/Immunofluorescence Staining/6d ctrl.png]

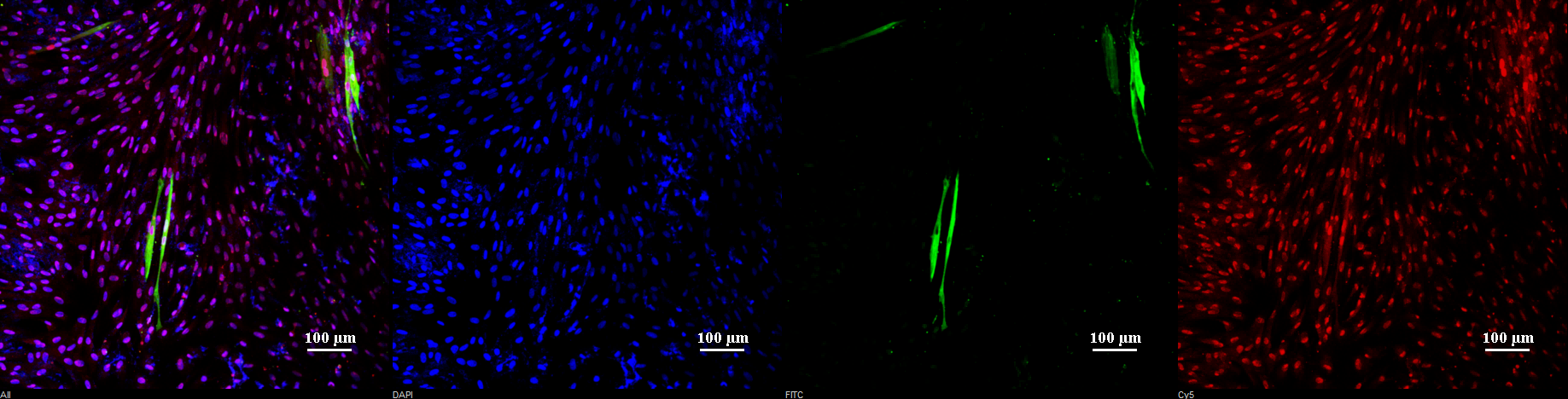

Supplement: Supplementary file 1 [file Data_Sheet_1.zip › Original Images/Immunofluorescence Staining/6d inhibitor.png]

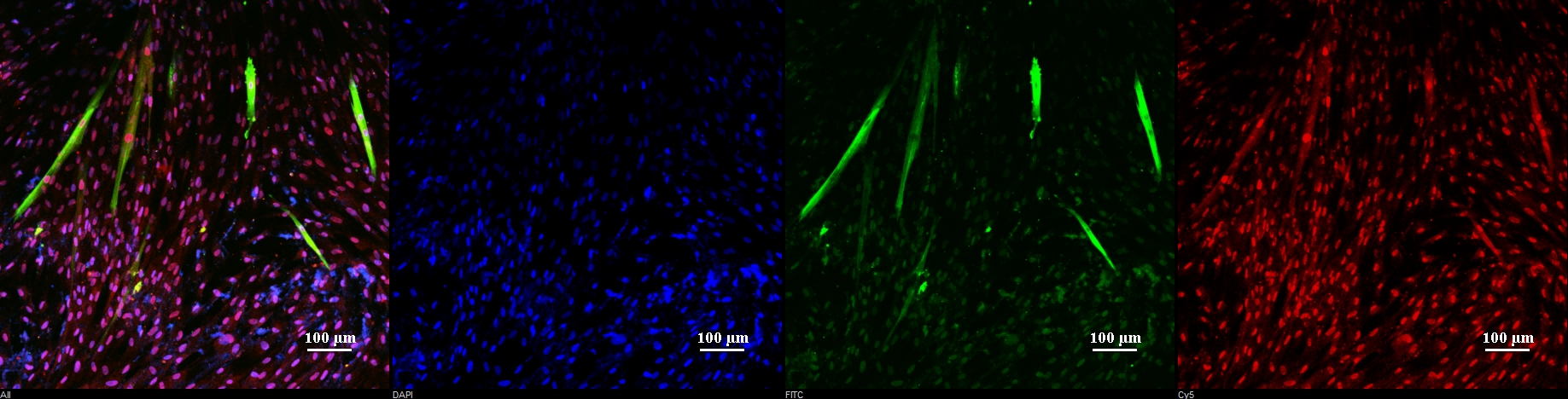

Supplement: Supplementary file 1 [file Data_Sheet_1.zip › Original Images/Immunofluorescence Staining/6d mimics.png]

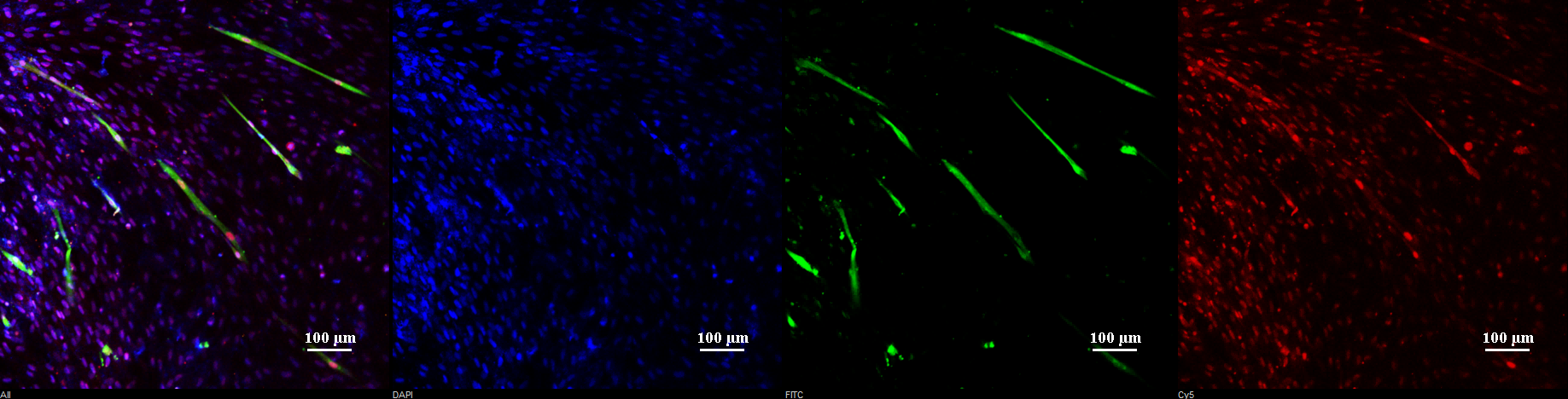

Supplement: Supplementary file 1 [file Data_Sheet_1.zip › Original Images/Immunofluorescence Staining/8d ctrl.png]

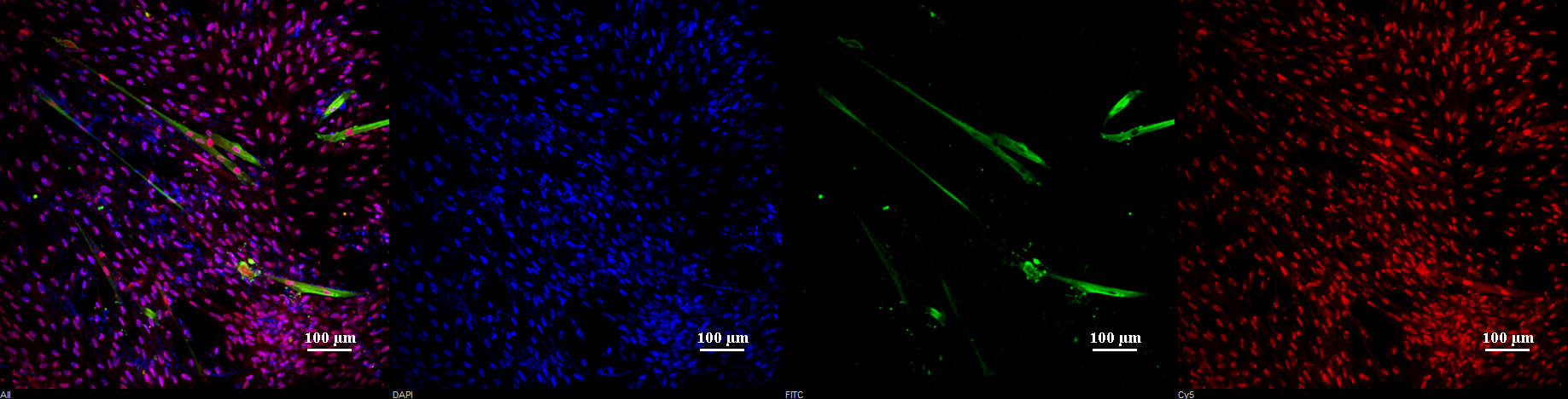

Supplement: Supplementary file 1 [file Data_Sheet_1.zip › Original Images/Immunofluorescence Staining/8d inhibitor.png]

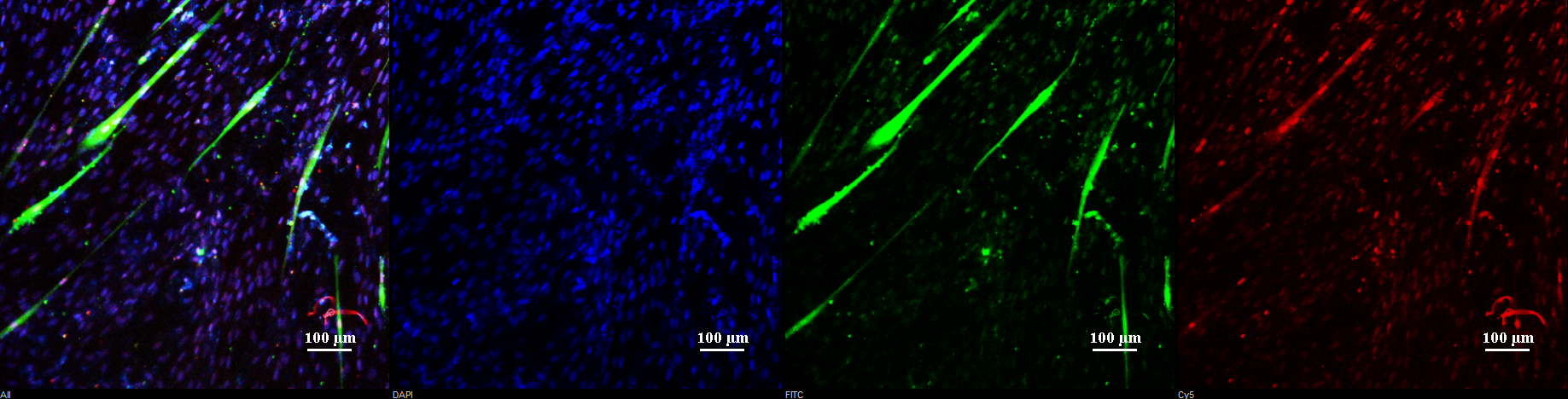

Supplement: Supplementary file 1 [file Data_Sheet_1.zip › Original Images/Immunofluorescence Staining/8d mimics.png]

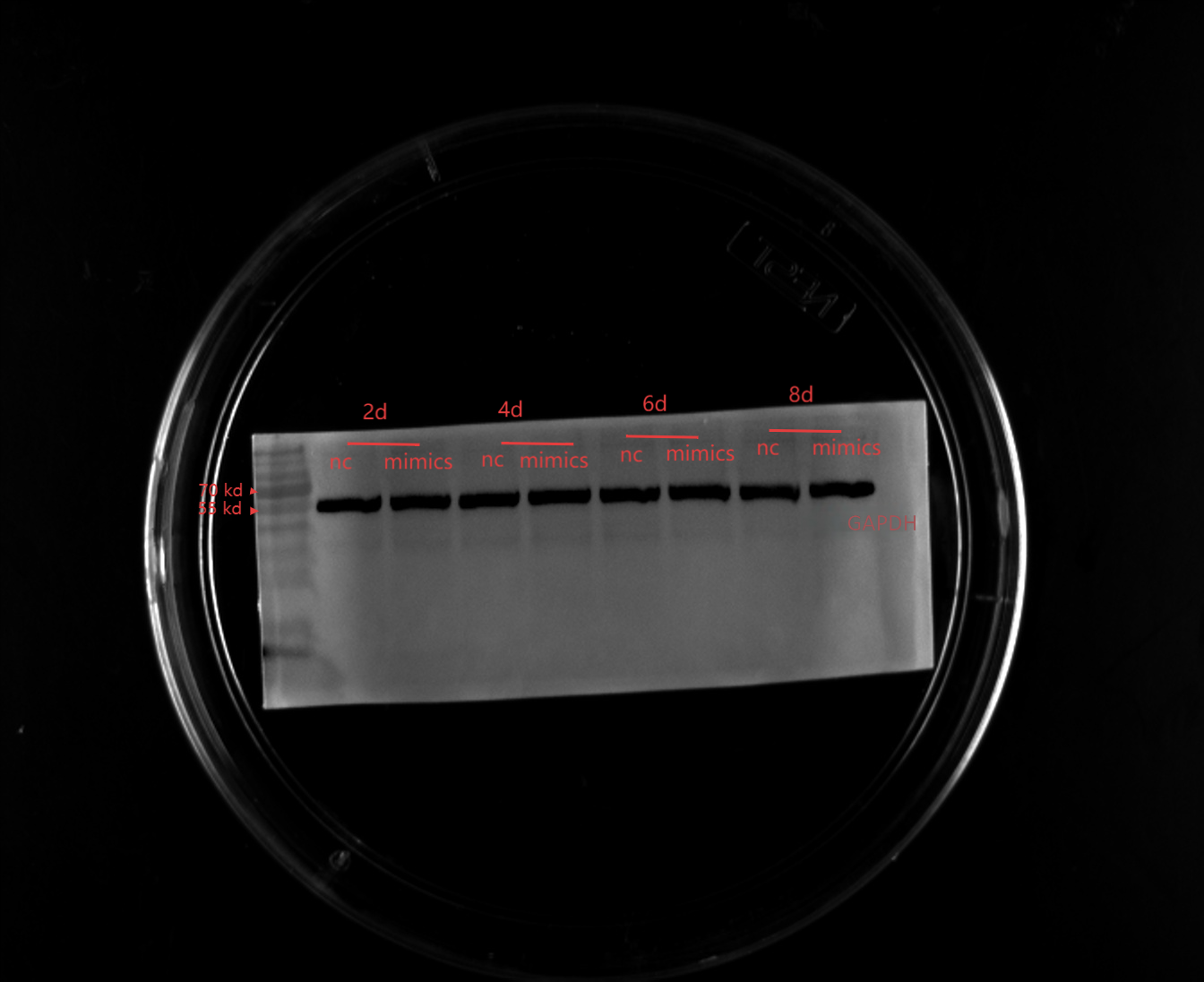

Supplement: Supplementary file 1 [file Data_Sheet_1.zip › Original Images/WB/Fig 1 D GAPDH(2).png]

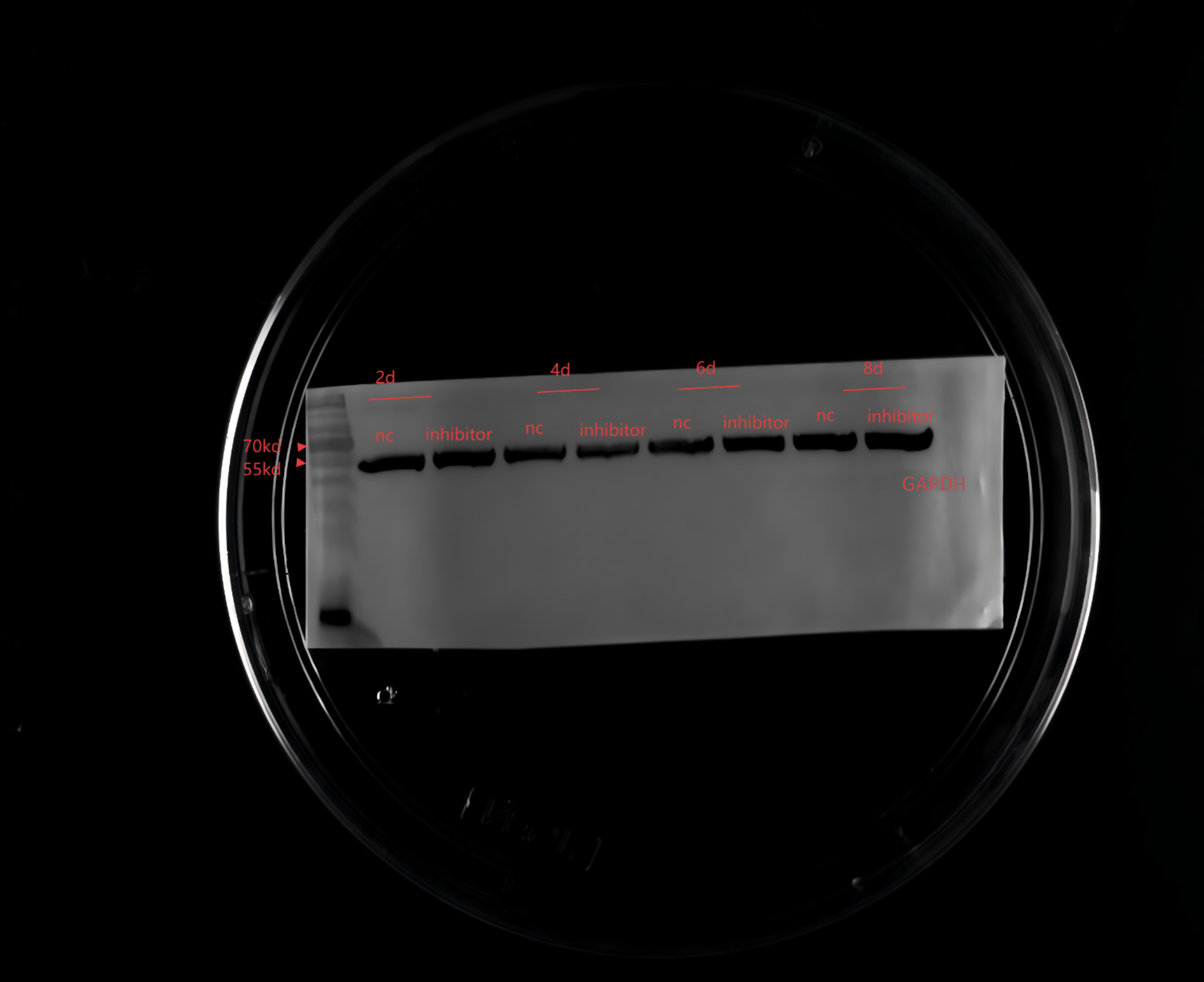

Supplement: Supplementary file 1 [file Data_Sheet_1.zip › Original Images/WB/Fig 2 D GAPDH(2).png]

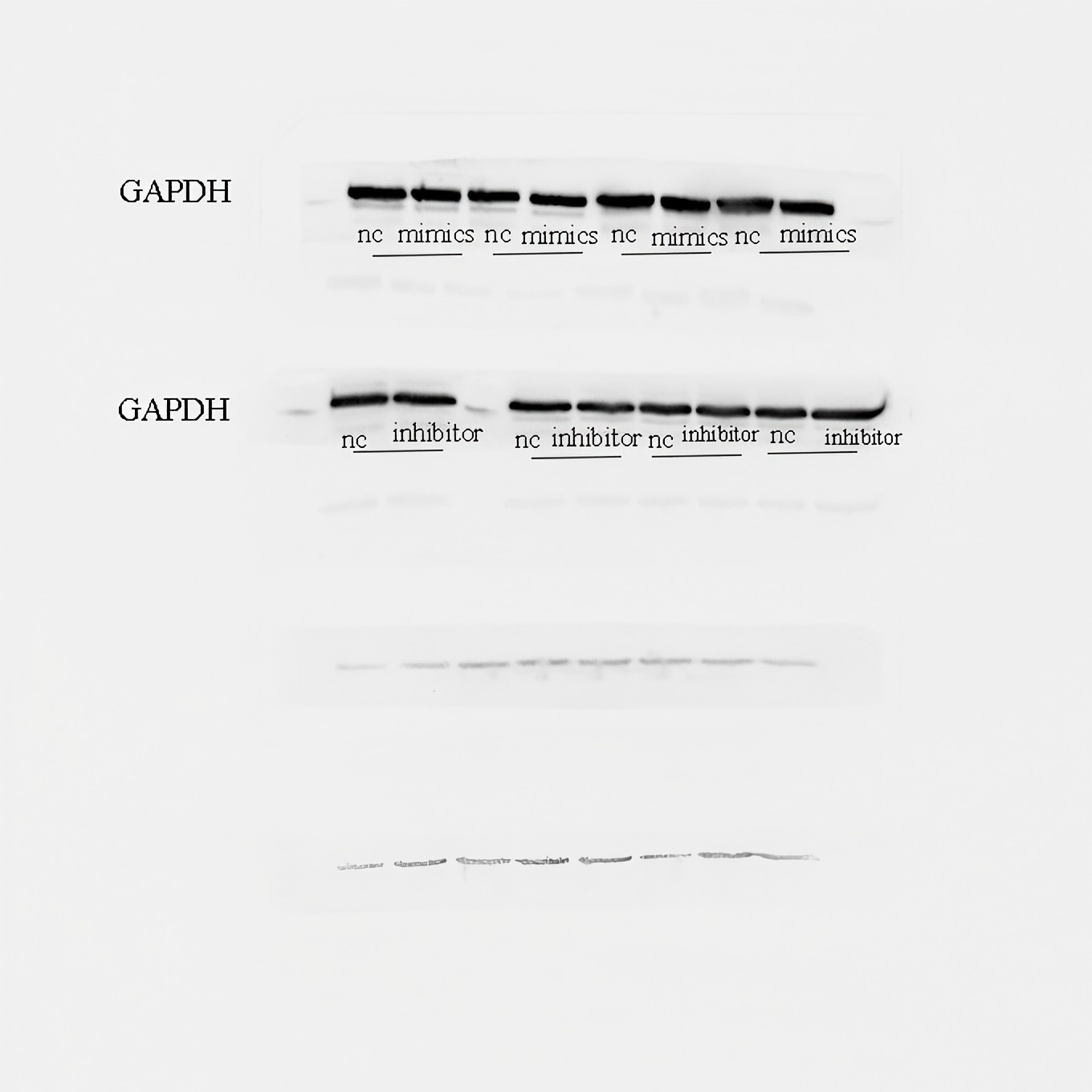

Supplement: Supplementary file 1 [file Data_Sheet_1.zip › Original Images/WB/Fig1 and 2 Gapdh.png]

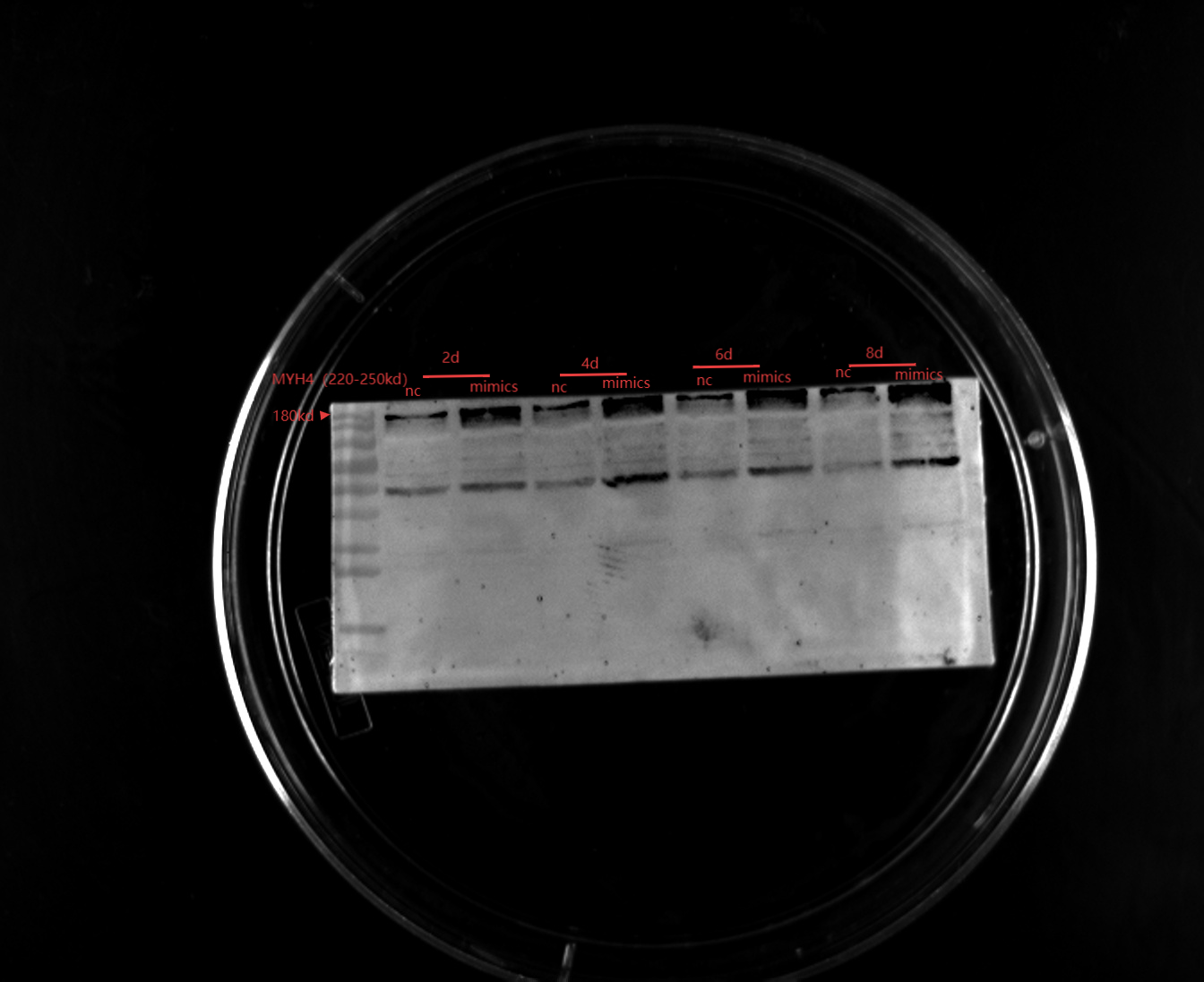

Supplement: Supplementary file 1 [file Data_Sheet_1.zip › Original Images/WB/Fig1 D MYH4(2).png]

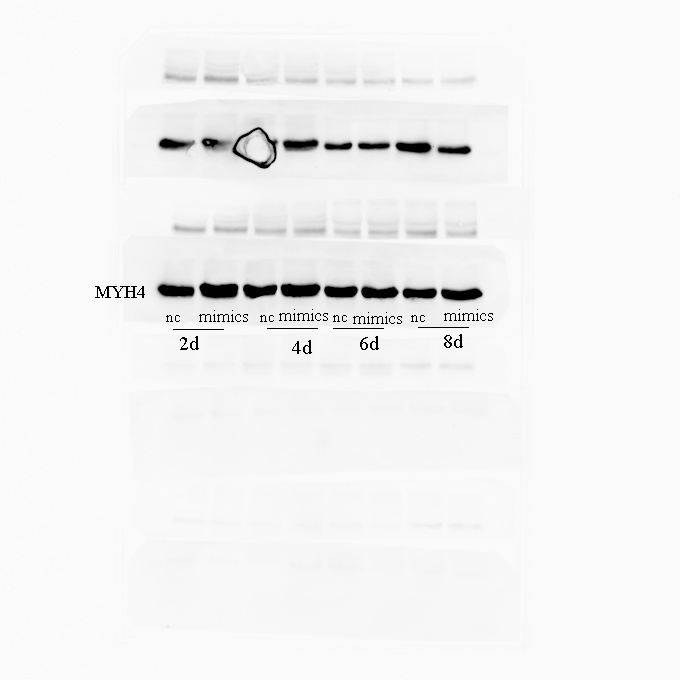

Supplement: Supplementary file 1 [file Data_Sheet_1.zip › Original Images/WB/Fig1 D MYH4.png]

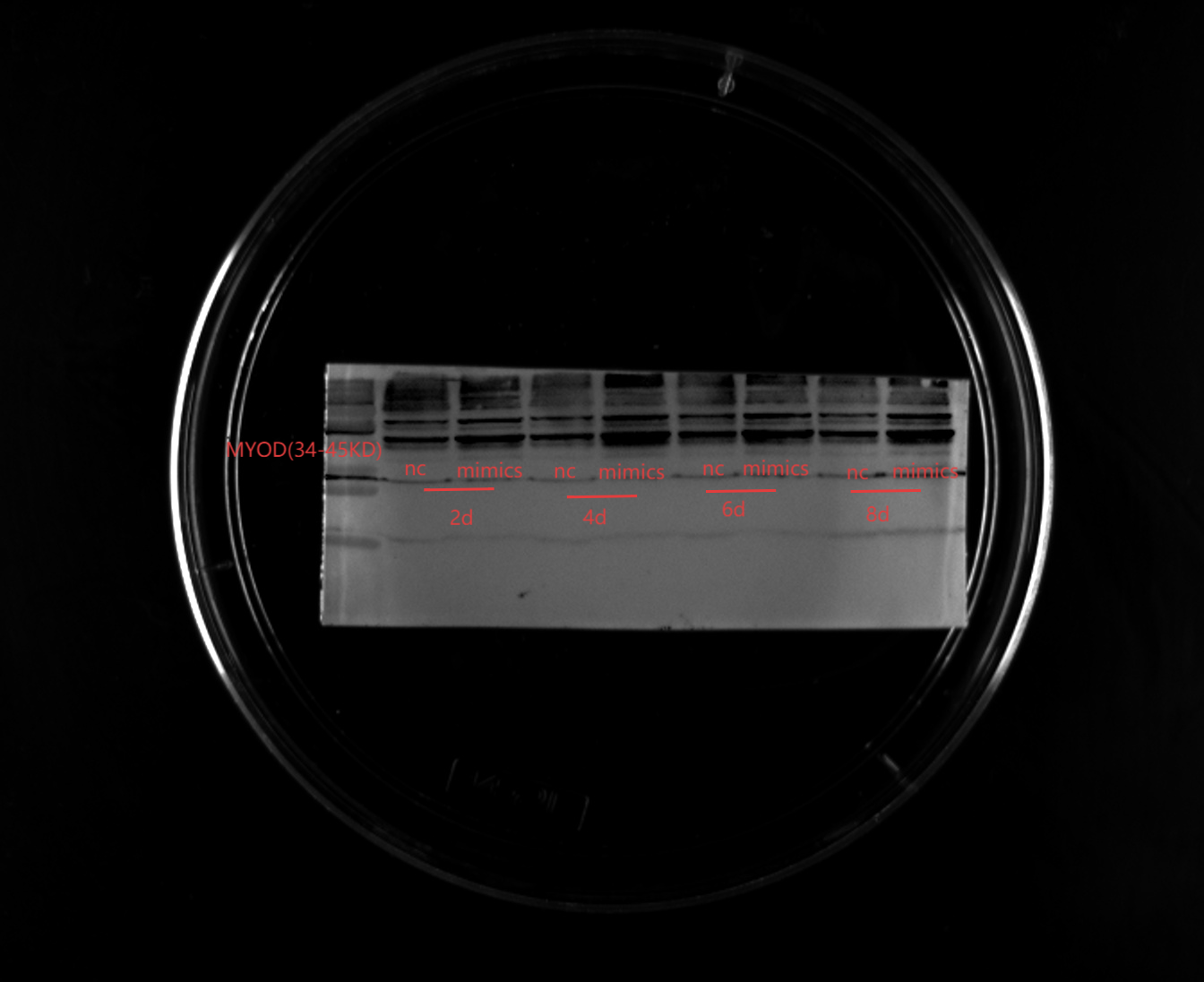

Supplement: Supplementary file 1 [file Data_Sheet_1.zip › Original Images/WB/Fig1 D MYOD(2).png]

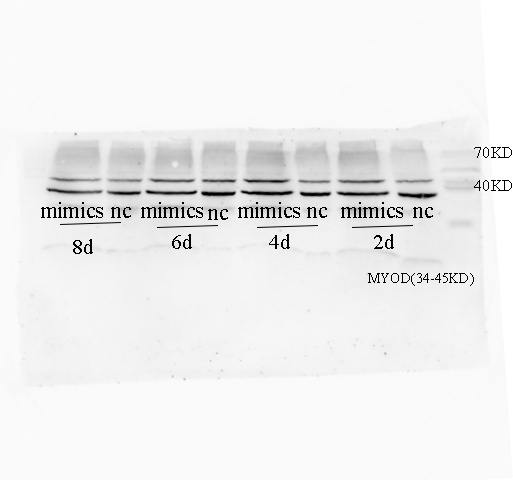

Supplement: Supplementary file 1 [file Data_Sheet_1.zip › Original Images/WB/Fig1 D MYOD.png]

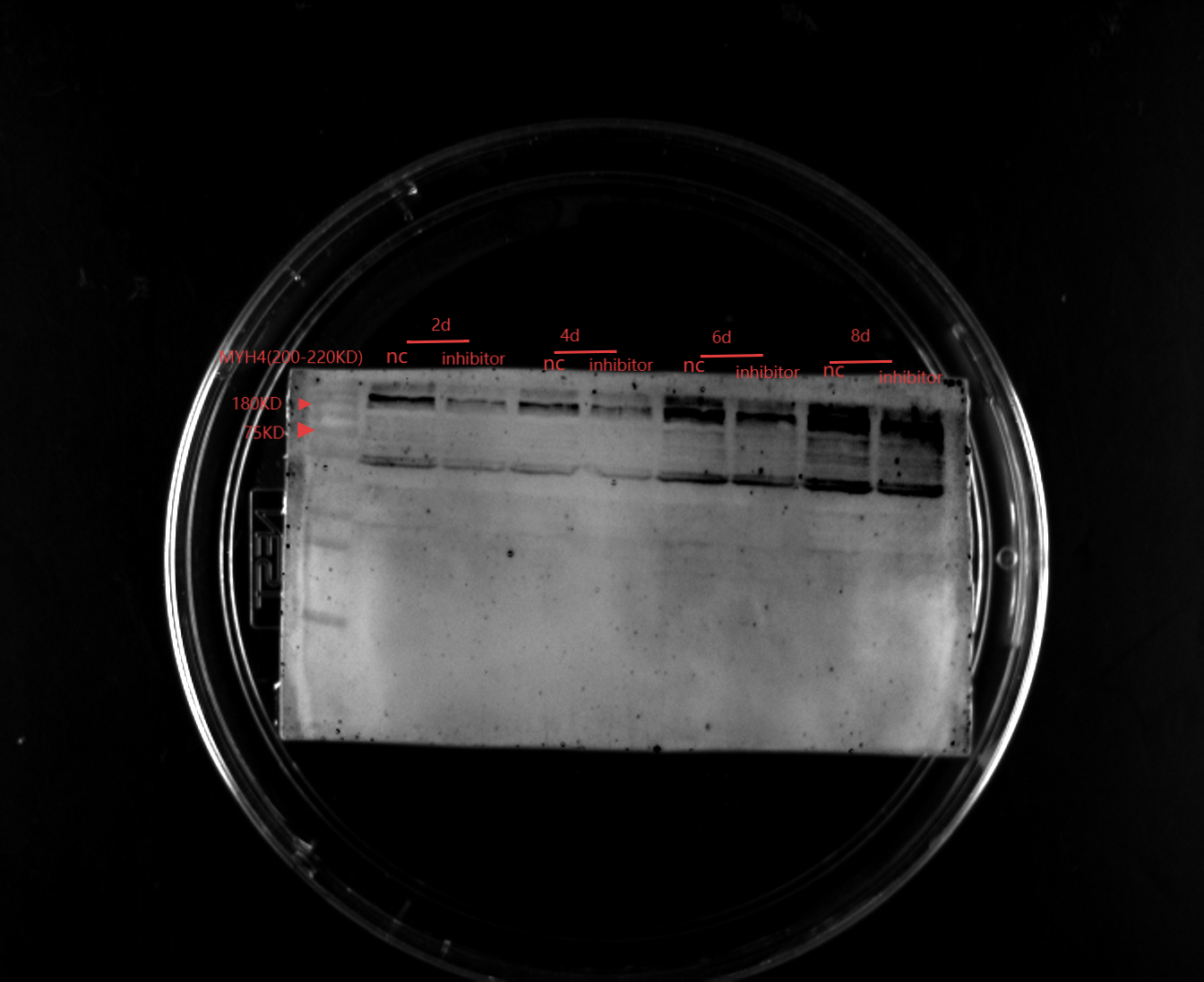

Supplement: Supplementary file 1 [file Data_Sheet_1.zip › Original Images/WB/Fig2 D MYH4(2).png]

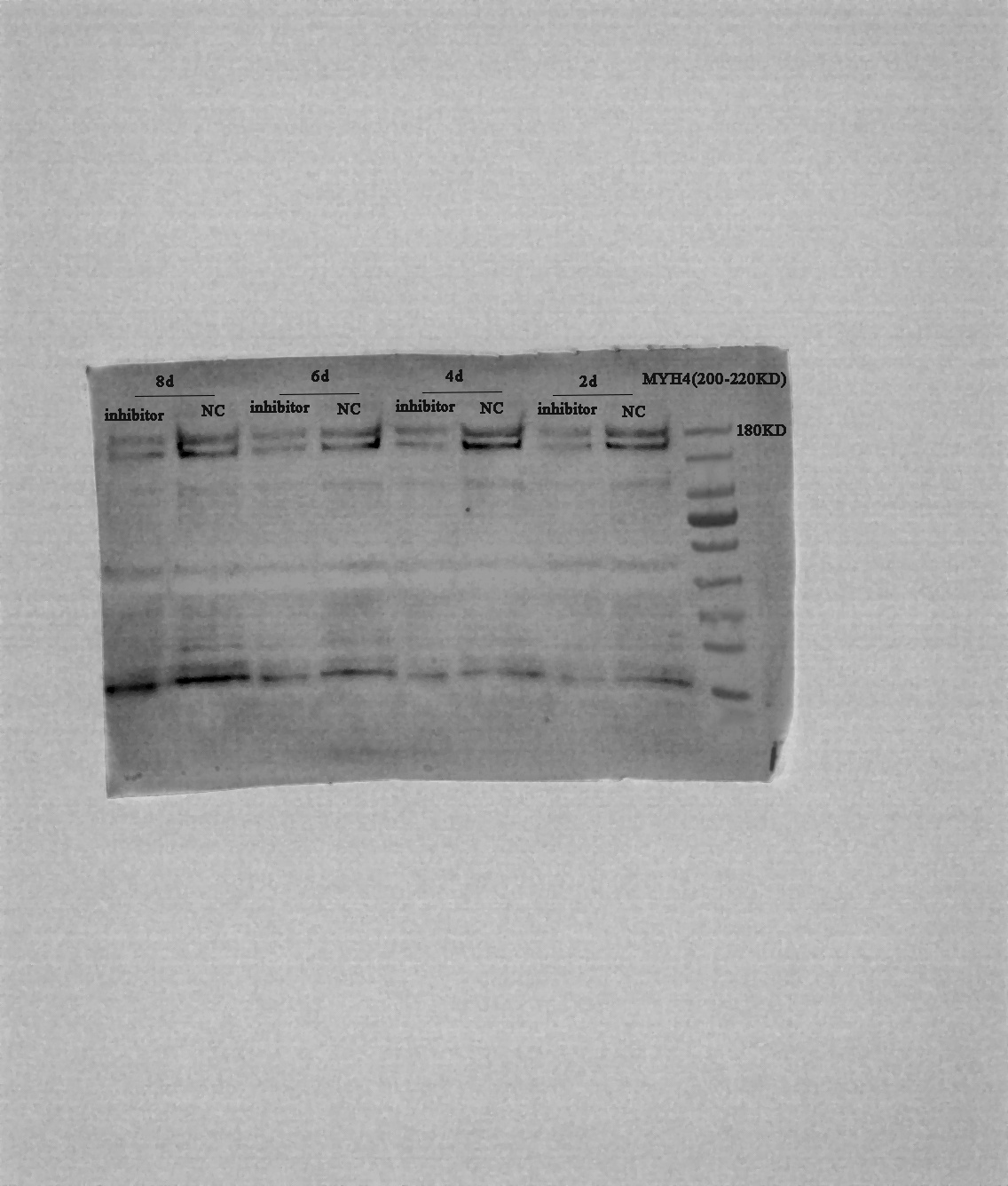

Supplement: Supplementary file 1 [file Data_Sheet_1.zip › Original Images/WB/Fig2 D MYH4.png]

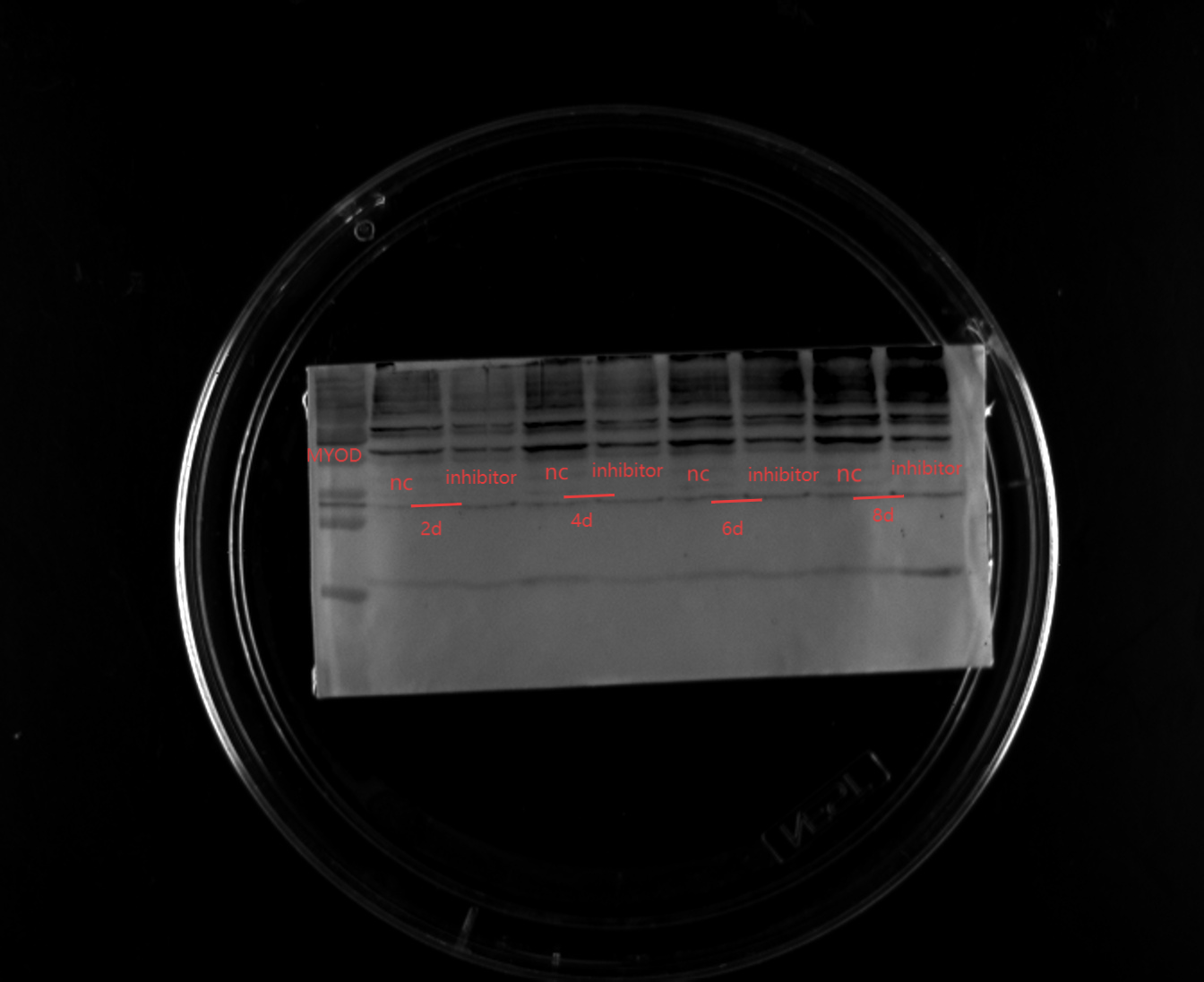

Supplement: Supplementary file 1 [file Data_Sheet_1.zip › Original Images/WB/Fig2 D MYOD(2).png]

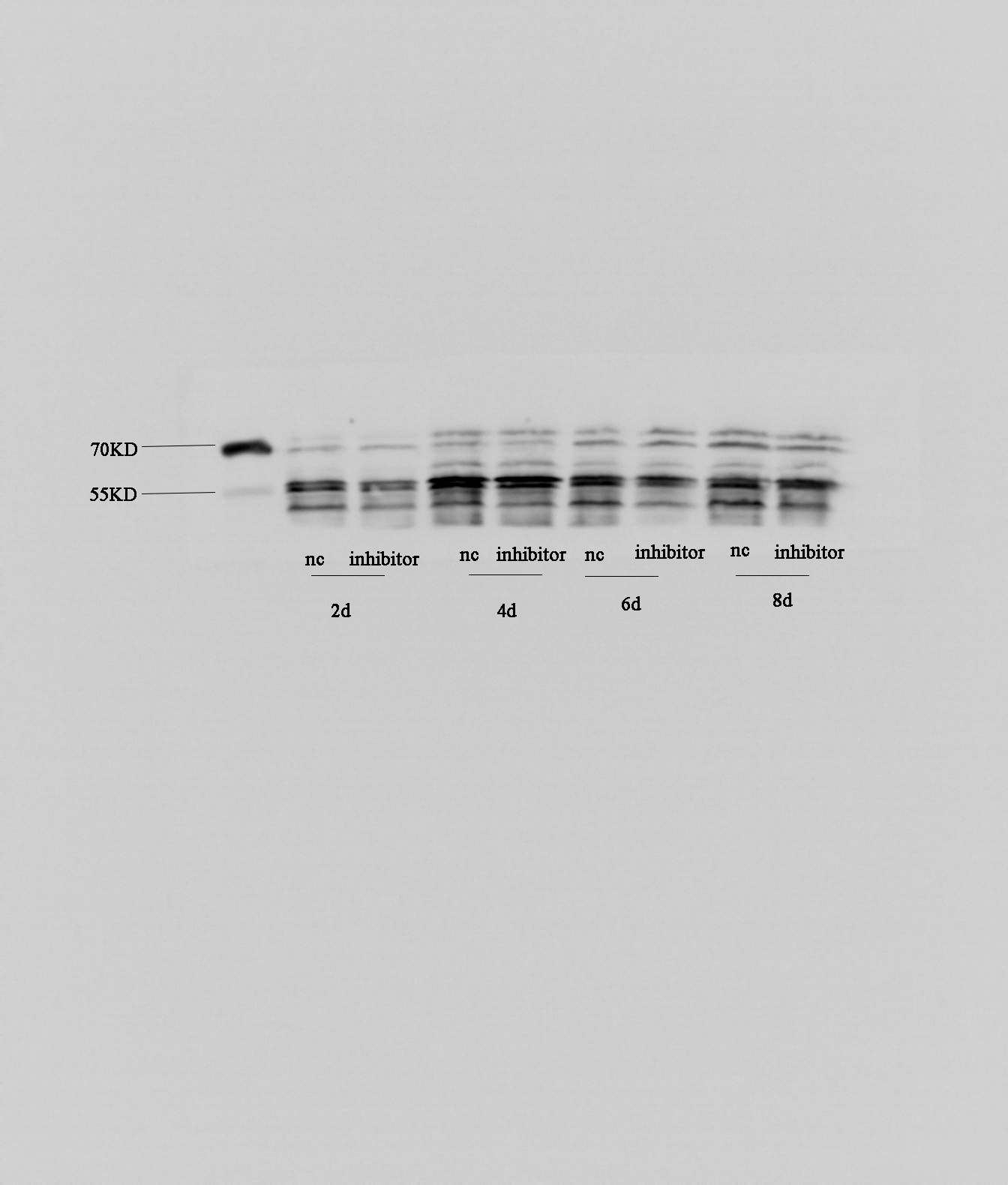

Supplement: Supplementary file 1 [file Data_Sheet_1.zip › Original Images/WB/Fig2 D MYOD.png]

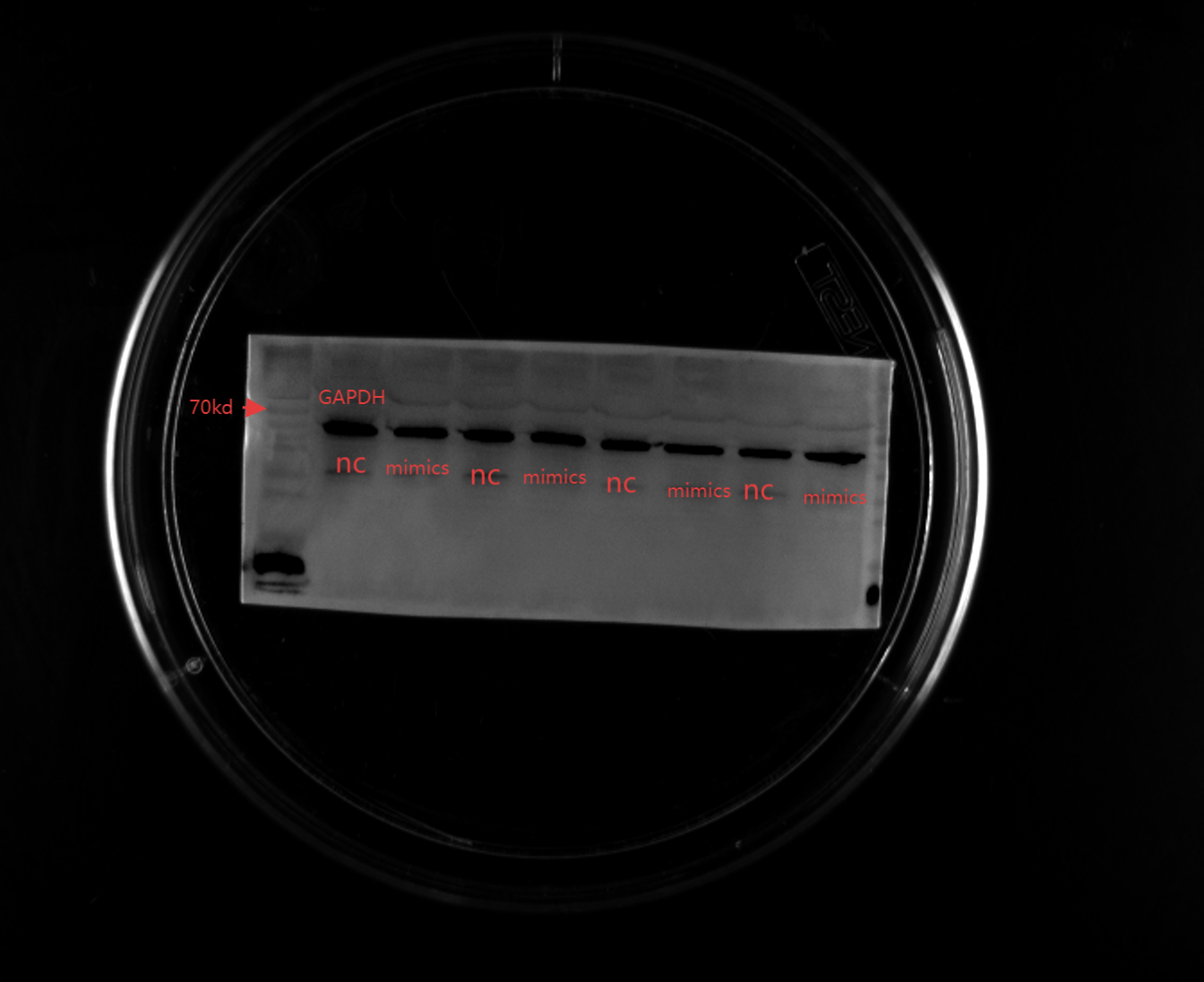

Supplement: Supplementary file 1 [file Data_Sheet_1.zip › Original Images/WB/Fig4 C GAPDH(2).png]

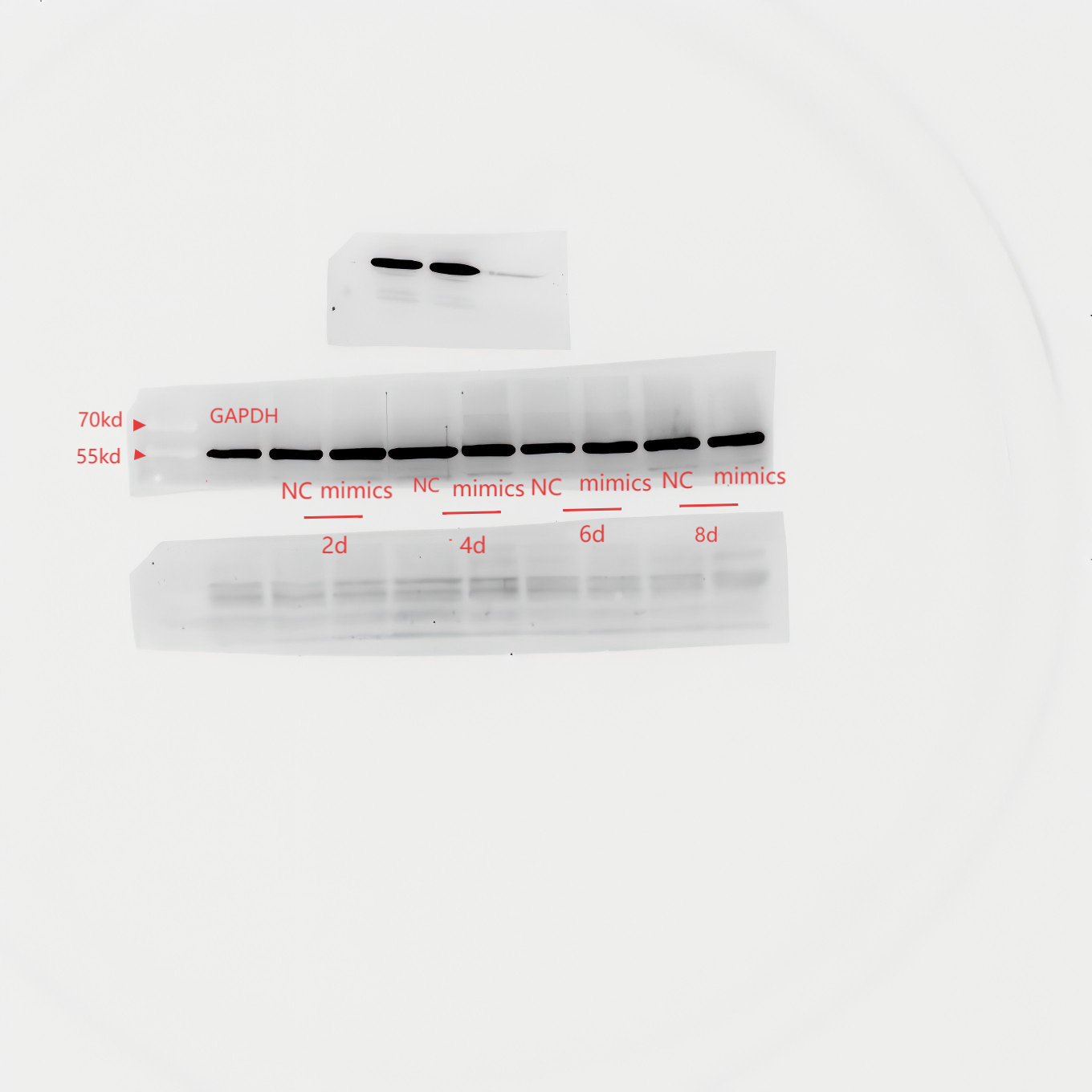

Supplement: Supplementary file 1 [file Data_Sheet_1.zip › Original Images/WB/Fig4 C GAPDH.png]

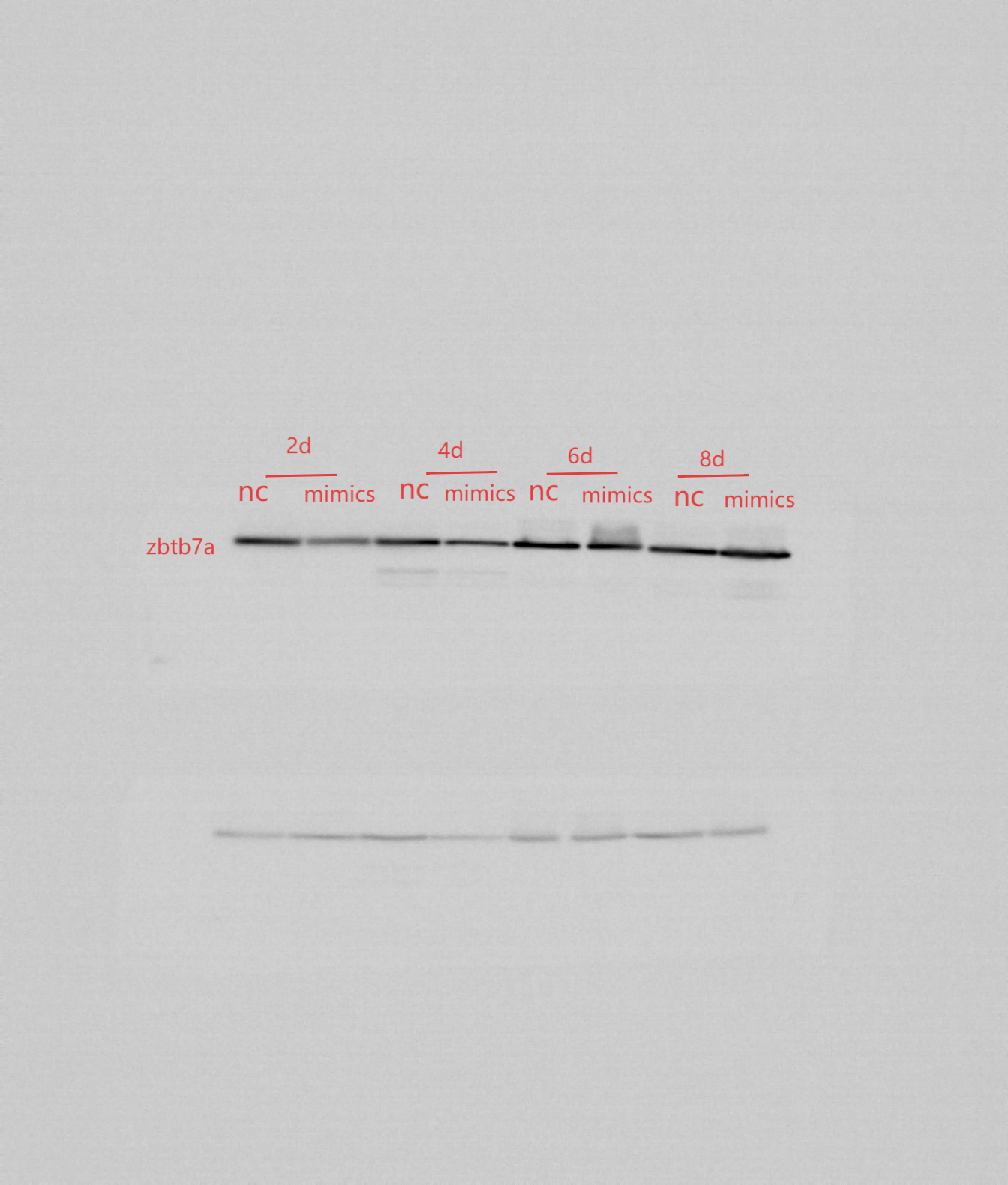

Supplement: Supplementary file 1 [file Data_Sheet_1.zip › Original Images/WB/Fig4 C ZBTB7A(2).png]

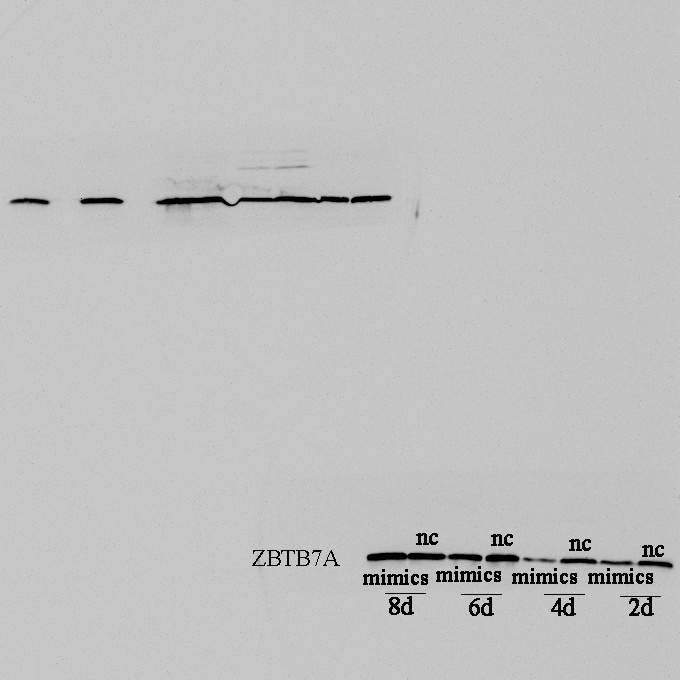

Supplement: Supplementary file 1 [file Data_Sheet_1.zip › Original Images/WB/Fig4 C ZBTB7A.png]

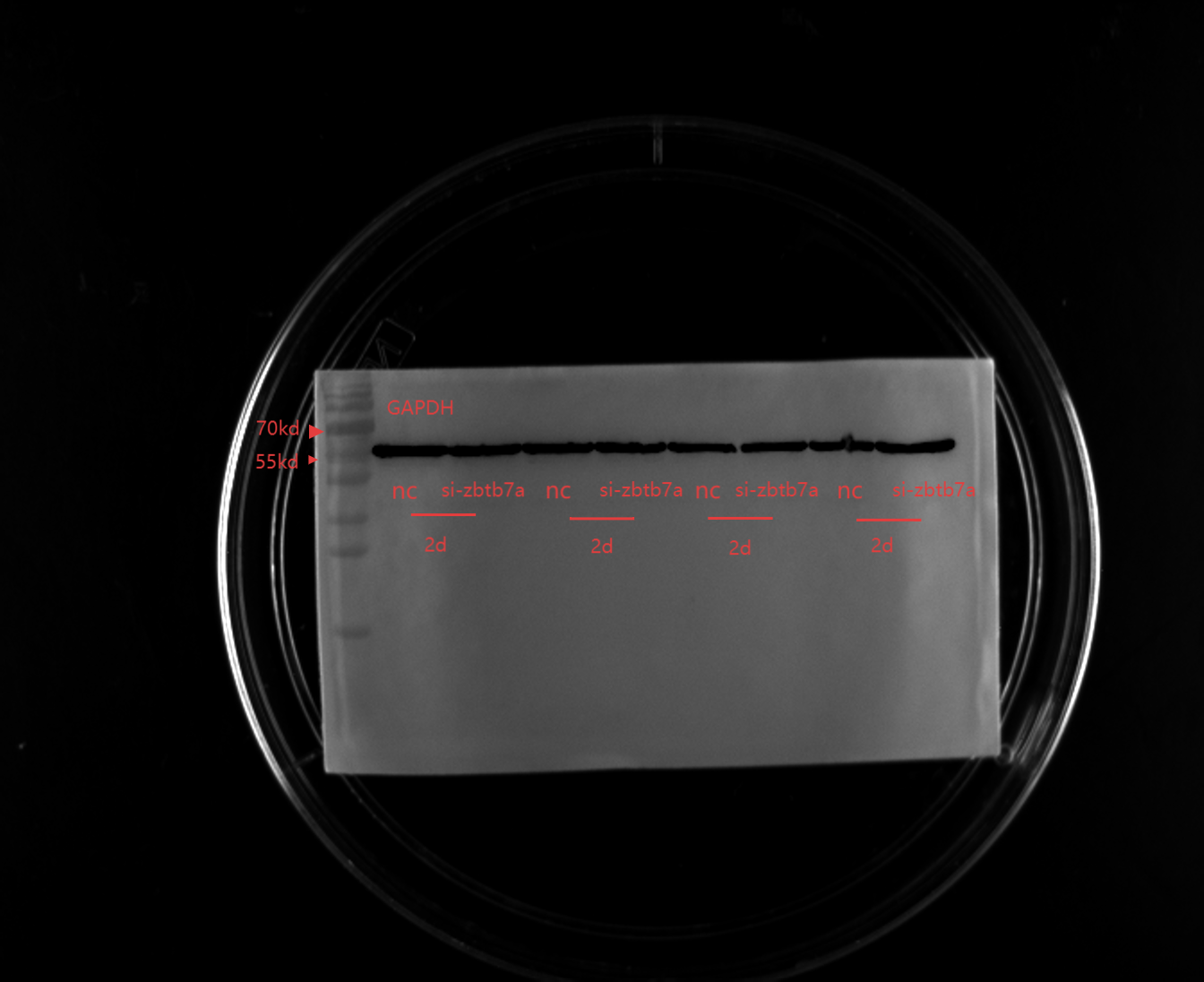

Supplement: Supplementary file 1 [file Data_Sheet_1.zip › Original Images/WB/Fig5 B GAPDH (2).png]

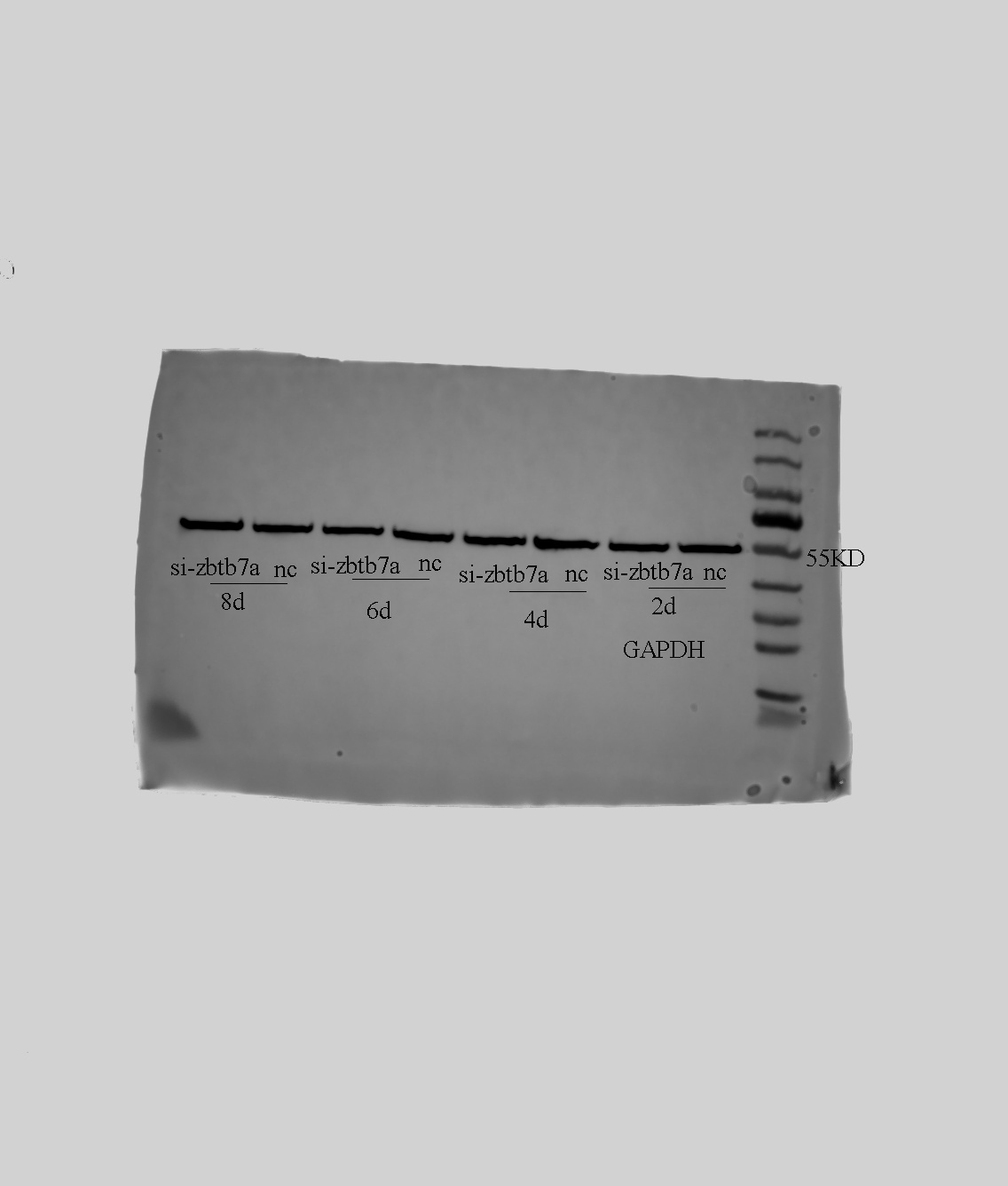

Supplement: Supplementary file 1 [file Data_Sheet_1.zip › Original Images/WB/Fig5 B GAPDH(1).png]

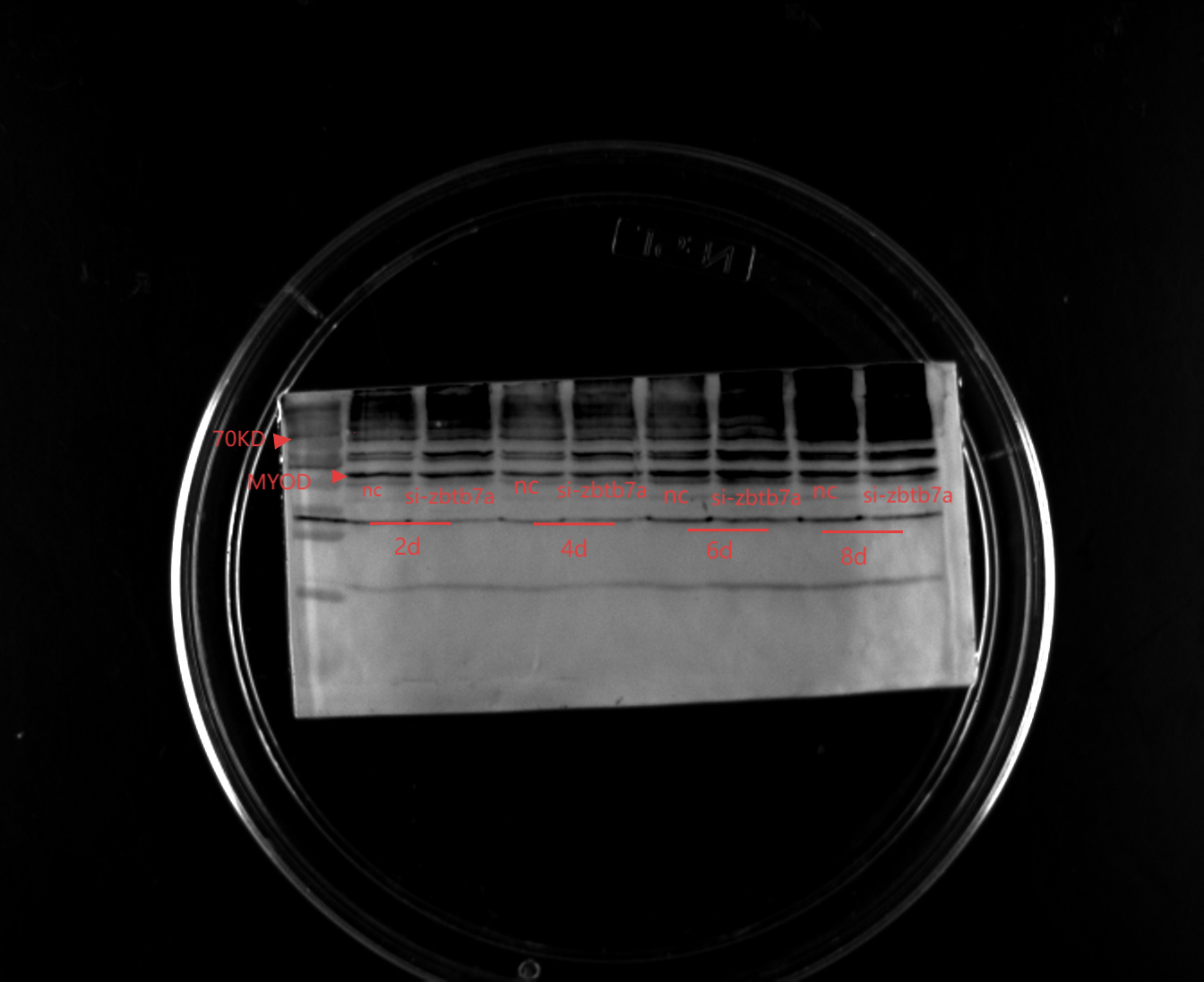

Supplement: Supplementary file 1 [file Data_Sheet_1.zip › Original Images/WB/Fig5 B MYOD(2).png]

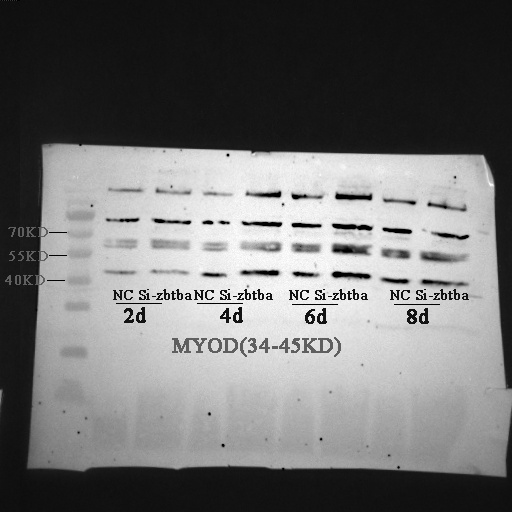

Supplement: Supplementary file 1 [file Data_Sheet_1.zip › Original Images/WB/Fig5 B MYOD.png]
